# Structural basis of transcription regulation by CNC family transcription factor, Nrf2

**DOI:** 10.1093/nar/gkac1102

**Published:** 2022-12-01

**Authors:** Toru Sengoku, Masaaki Shiina, Kae Suzuki, Keisuke Hamada, Ko Sato, Akiko Uchiyama, Shunsuke Kobayashi, Asako Oguni, Hayato Itaya, Kota Kasahara, Hirotomo Moriwaki, Chiduru Watanabe, Teruki Honma, Chikako Okada, Shiho Baba, Tsutomu Ohta, Hozumi Motohashi, Masayuki Yamamoto, Kazuhiro Ogata

**Affiliations:** Department of Biochemistry, Yokohama City University Graduate School of Medicine, Yokohama 236-0004, Japan; Department of Biochemistry, Yokohama City University Graduate School of Medicine, Yokohama 236-0004, Japan; Department of Biochemistry, Yokohama City University Graduate School of Medicine, Yokohama 236-0004, Japan; Department of Biochemistry, Yokohama City University Graduate School of Medicine, Yokohama 236-0004, Japan; Department of Biochemistry, Yokohama City University Graduate School of Medicine, Yokohama 236-0004, Japan; Department of Biochemistry, Yokohama City University Graduate School of Medicine, Yokohama 236-0004, Japan; Department of Biochemistry, Yokohama City University Graduate School of Medicine, Yokohama 236-0004, Japan; Department of Biochemistry, Yokohama City University Graduate School of Medicine, Yokohama 236-0004, Japan; College of Life Sciences, Ritsumeikan University, Kusatsu 525-8577, Japan; College of Life Sciences, Ritsumeikan University, Kusatsu 525-8577, Japan; RIKEN Center for Biosystems Dynamics Research, Yokohama 230-0045, Japan; RIKEN Center for Biosystems Dynamics Research, Yokohama 230-0045, Japan; JST PRESTO, Yokohama 230-0045, Japan; RIKEN Center for Biosystems Dynamics Research, Yokohama 230-0045, Japan; Department of Biochemistry, Yokohama City University Graduate School of Medicine, Yokohama 236-0004, Japan; Department of Biochemistry, Yokohama City University Graduate School of Medicine, Yokohama 236-0004, Japan; Department of Physical Therapy, Faculty of Health and Medical Sciences, Tokoha University, Hamamatsu 431-2102, Japan; Department of Gene Expression Regulation, Institute of Development, Aging, and Cancer, Tohoku University, Sendai 980-8575, Japan; Department of Integrative Genomics, Tohoku Medical Megabank Organization, Tohoku University, Sendai 980-8575, Japan; Department of Biochemistry, Yokohama City University Graduate School of Medicine, Yokohama 236-0004, Japan

## Abstract

Several basic leucine zipper (bZIP) transcription factors have accessory motifs in their DNA-binding domains, such as the CNC motif of CNC family or the EHR motif of small Maf (sMaf) proteins. CNC family proteins heterodimerize with sMaf proteins to recognize CNC–sMaf binding DNA elements (CsMBEs) in competition with sMaf homodimers, but the functional role of the CNC motif remains elusive. In this study, we report the crystal structures of Nrf2/NFE2L2, a CNC family protein regulating anti-stress transcriptional responses, in a complex with MafG and CsMBE. The CNC motif restricts the conformations of crucial Arg residues in the basic region, which form extensive contact with the DNA backbone phosphates. Accordingly, the Nrf2–MafG heterodimer has approximately a 200-fold stronger affinity for CsMBE than canonical bZIP proteins, such as AP-1 proteins. The high DNA affinity of the CNC–sMaf heterodimer may allow it to compete with the sMaf homodimer on target genes without being perturbed by other low-affinity bZIP proteins with similar sequence specificity.

## INTRODUCTION

The Cap’n’collar (CNC) family proteins are a group of the basic leucine zipper (bZIP) type transcription factors (TFs) that have essential roles in cell fate decisions and cellular responses to environmental stresses and stimulations (1,2). They form heterodimers with another group of bZIP TFs, small Maf (sMaf) proteins, via their leucine zipper regions. The resulting heterodimers recognize the CNC–sMaf binding elements (CsMBEs) (3), also called antioxidant responsive elements (AREs) or electrophile responsive elements (EpREs), and function as transcriptional activators or repressors, depending on whether the CNC subunit has a transactivation domain (4). sMaf proteins can form homodimers that preferentially bind to sequences called Maf-responsive elements (MAREs) and function as transcriptional repressors (5). The consensus sequences of CsMBEs and MAREs are similar (see below), and CNC–sMaf heterodimers and sMaf homodimers often bind to both elements, albeit with slightly different binding affinities. CNC–sMaf heterodimers and sMaf homodimers competitively bind to regulatory DNA elements with each other and with other bZIP proteins, and their relative abundances and affinities for the elements determine whether their target genes are transcribed (6–11).

While AP-1 proteins bind their target DNA only through their bZIP regions, the DNA-binding domains of CNC family and sMaf proteins contain the CNC motif and the extended homology region (EHR), respectively, which are located N-terminal to the canonical bZIP region. The CsMBE consensus sequence is the non-palindromic 11-bp (5′-[A/G]TGA[G/C]TCAGCT-3′), of which the [A/G]TGA and TCAGCT half sites are recognized by the CNC and sMaf proteins, respectively. On the other hand, the MARE consensus sequence is the palindromic 13-bp (5′-AGCTGA[G/C]TCAGCT-3′), of which two 5′-TCAGCT-3′ half sites are bound by two protomers of the sMaf homodimer. Both CsMBE and MARE contain the central 7-mer element (5′-TGA[G/C]TCA-3′), which is known as the 12-*O*-tetradecanoylphorbol 13-acetate-responsive element (TRE), the target of AP-1 proteins (12). Crystallographic analyses have revealed how MafG (a sMaf protein) forms a homodimer and uniquely recognizes the MARE element (13). However, the mechanisms through which CNC family proteins recognize the CsMBE sequence and the involvement of their CNC motifs remain unclear.

Nuclear factor erythroid 2-like factor (Nrf2/NFE2L2) is a member of the CNC family and plays a central role in cellular defense mechanisms against oxidative and electrophilic stresses (14). Under unstressed conditions, Nrf2 is constantly ubiquitinated in the cytoplasm by a cellular stress sensor protein, Kelch-like ECH-associated protein 1 (Keap1), and is degraded by the proteasome (15,16). Oxidative and electrophilic reagents covalently modify the specific Cys residues of Keap1 (17), which leads to the release and stabilization of Nrf2. Stabilized Nrf2 then translocates to the nucleus and forms heterodimers with sMaf proteins, thereby functioning as a transcriptional activator and upregulating the target genes involved in anti-stress responses. Small-molecule drugs that stabilize Nrf2 have been developed to treat several chronic diseases that cause oxidative stress, such as multiple sclerosis and diabetic nephropathy (18). Nrf2 is also known to be aberrantly activated in many cancers (19), possibly upregulating the cytoprotective genes that favor the growth of cancer cells. A recent pan-cancer large-scale analysis identified that the Nrf2-Keap1 pathway is one of the major oncogenic signaling pathways (20). These results suggest that precise control of the Nrf2-dependent transcriptional program is essential for normal cellular functions.

To understand the transcription regulation mechanism of CNC family proteins, we conducted structural, biochemical, and computational analyses of Nrf2. The co-crystal structures of Nrf2–MafG-CsMBE complexes show that the CNC motif stabilizes the conformation of Arg residues in the basic region in contact with DNA phosphates. As a result, Nrf2 forms extensive contact with DNA and recognizes the backbone phosphates up to four nucleotides outside of the core TRE element. Consistently, the Nrf2–MafG heterodimer and the MafG homodimer bind to CsMBE with an affinity that is approximately 200-fold stronger than that of typical AP-1 factors. Deleting the CNC motif significantly reduced the affinity of the heterodimer for CsMBE, resulting in defective transcriptional activation by Nrf2. We propose that the high affinity of CNC–sMaf heterodimers and sMaf homodimers enables the precise and timely regulation of their target genes through their competitive actions, depending on their relative abundance, without being perturbed by AP-1 proteins recognizing similar central core DNA elements.

## MATERIALS AND METHODS

The sequences of the recombinant proteins and DNA used in this study are shown in Supplementary Table S1.

### Protein expression and purification

For crystallization, a modified pET14b plasmid encoding human Nrf2 fragment (residues 452–560) with an N-terminal hexahistidine (His6) tag cleavable with the HRV 3C protease was introduced into *Escherichia coli* BL21 (DE3), and the cells were grown in terrific broth supplemented with 300 μg/ml ampicillin at 37°C. Protein expression was induced by adding isopropyl β-D-1-thiogalactopyranoside (IPTG) at a final concentration of 1 mM at an optical density of 0.8, and the culture was continued for 3 h at 37°C. The harvested cells were suspended in 50 mM Tris–HCl pH 8.0, 0.5 mM EDTA, 100 mM NaCl, 1 mM DTT, 0.125 mM phenylmethylsulfonyl fluoride (PMSF), disrupted with a pressure-type homogenizer, and the lysate was centrifuged at 20 000 × g. The supernatant was subjected to the phosphocellulose P11 column (Whatman) and eluted using a linear gradient from 100 mM to 1.5 M NaCl in 50 mM Tris–HCl pH 8.0, 0.5 mM EDTA. The eluted protein was then purified using a HisTrap HP column (GE Healthcare) with a linear gradient from 10 mM to 500 mM imidazole in 20 mM potassium phosphate pH 6.5, 100 mM NaCl, followed by affinity tag removal by incubating with the HRV 3C protease overnight at 4°C. The protein was further purified using a HiTrap SP column (GE Healthcare) with a linear gradient from 0 to 1 M NaCl in 50 mM HEPES-Na pH 7.5, 0.5 mM EDTA, and using a Superdex 75 column (GE Healthcare) in 50 mM HEPES-Na pH 7.5, 0.5 mM EDTA, 100 mM NaCl. Finally, the protein was dialyzed against distilled water containing 10 mM of DTT, concentrated using Vivaspin (Sartorius), flash-frozen with liquid nitrogen, and stored at −80°C. The constructs for Nrf2 mutants were created using the QuikChange Site-Directed Mutagenesis Kit (Agilent Technology) or the Primestar Mutagenesis Kit (Takara), and the mutant proteins were expressed and purified similarly. A longer Nrf2 fragment (residues 182–560) and its mutants used for the electrophoretic mobility shift assay (EMSA) were also expressed and purified similarly, but their His6 tag was not removed.

For the human MafG expression used for crystallization, we utilized the *plating* method (21) to overcome the high toxicity of human MafG protein to host cells. Briefly, a single colony of BL21 (DE3) cells transformed with pAR2156-MafG (residues 21–123, without any tag) was suspended in 200 ml of distilled water, and the cell suspension was plated on several LB plates containing 50 μg/ml ampicillin and incubated at 37°C overnight. All colonies were scrapped and transferred to 400 ml of LB medium containing 50 μg/ml ampicillin. The resulting medium was cultured at 37°C for 1 h, and protein expression was induced by adding IPTG at a final concentration of 1 mM; the culture was continued for 3 h. The harvested cells were suspended in 50 mM Tris–HCl pH 8.0, 20 mM NaCl, 0.5 mM EDTA, 1 mM DTT, disrupted with a pressure-type homogenizer, and the lysate was centrifuged at 20 000 × g. The supernatant was subjected to a HiTrap Heparin column and eluted using a linear gradient from 100 mM to 1 M NaCl in 20 mM Tris–HCl pH 8.0. 0.5 mM EDTA. The eluted protein was further purified by a Superdex 75 column in 20 mM Tris–HCl pH 8.0, 100 mM NaCl, 0.5 mM EDTA, 1 mM DTT. Finally, the protein was dialyzed against distilled water containing 10 mM of DTT, concentrated using Vivaspin, flash-frozen with liquid nitrogen, and stored at −80°C.

For the EMSA, a modified pET14b plasmid encoding human MafG fragment (residues 21–123) with an N-terminal His6 tag cleavable with the HRV 3C protease was introduced into E.coli BL21(DE3)-RIL, and the cells were grown in terrific broth supplemented with 300 ug/ml ampicillin at 37°C. Protein expression was induced by adding IPTG at a final concentration of 1 mM at an optical density of 1.0, and the culture was continued for 3 h at 37°C. The harvested cells were suspended in 50 mM Tris–HCl pH 8.0, 0.5 mM EDTA, 100 mM NaCl, 1 mM DTT, 0.125 mM PMSF, disrupted with a pressure-type homogenizer, and the lysate was centrifuged at 20,000 × g. The supernatant was subjected to the phosphocellulose P11 column and eluted using a linear gradient from 100 mM to 2.0 M NaCl in 50 mM Tris–HCl pH 8.0, 0.5 mM EDTA. The eluted protein was then purified using a HisTrap HP column with a linear gradient from 50 mM to 2.0 M imidazole in 20 mM potassium phosphate pH 6.5, 50 mM NaCl, followed by affinity tag removal by incubating with HRV 3C protease overnight at 4°C. The protein was further purified using a Superdex 75 column in 50 mM HEPES-Na pH 7.5, 0.5 mM EDTA, 150 mM NaCl. Finally, the His tag removed protein was dialyzed against distilled water containing 10 mM of DTT, concentrated using Vivaspin, flash-frozen with liquid nitrogen, and stored at −80°C.

A modified pET14b plasmid encoding human c-Fos fragment (residues 111–207) with an N-terminal His6 tag cleavable with the HRV 3C protease was introduced into *E. coli* BL21(DE3)-RIL, and the cells were grown in terrific broth supplemented with 300 ug/ml ampicillin, 30 ug/ml chloramphenicol, and 1% glucose at 37°C. The harvested cells were suspended in 50 mM Tris–HCl pH 8.0, 0.5 mM EDTA, 100 mM NaCl, 2 mM DTT, 0.125 mM PMSF and disrupted with a pressure-type homogenizer. The lysate was centrifuged at 20,000 × g. Precipitated c-Fos protein was solubilized in 50 mM Tris–HCl (pH 8.0), 6 M guanidine-HCl. The protein was then purified using a HisTrap HP column with a linear gradient from 0 mM to 500 mM imidazole in 50 mM Tris–HCl pH 8.0, 6 M guanidine-HCl, 10 mM NaCl, 1 mM DTT, and dialyzed against 25 mM MES-Na pH 6.0, 1.4 mM 2-mercaptoethanol. Finally, the protein was concentrated using Vivaspin, flash-frozen with liquid nitrogen, and stored at −80°C. Human c-Jun fragment (residues 237–331) was also expressed and purified similarly.

### DNA preparation for the structural study

Single-stranded DNA fragments purified by HPLC were purchased from FASMAC and annealed by heating at 98°C for 20 s and cooling down to 4°C at a rate of −6°C/min. To remove single-stranded DNA, the annealed DNA was subjected to hydroxy apatite chromatography, washed with 10 mM potassium phosphate buffer (pH 6.5), and eluted with a linear gradient of potassium phosphate buffer (pH 6.5) from 10 to 500 mM. The eluted DNA was concentrated using Vivaspin, dialyzed against water, and stored at −20°C.

### Crystallization and structure determination

For the crystallization of Nrf2–MafG–DNA complexes, purified Nrf2, MafG and DNA were mixed at a molar ratio of 1:1:1, and the total concentration of the complex was adjusted to 10 mg/ml (3.7 mg/ml of Nrf2, 3.4 mg/ml of MafG and 2.9 mg/ml of DNA) in 33 mM DTT. The formation of the heterodimer-DNA complex was carefully monitored with component titration and non-denaturing polyacrylamide gel electrophoresis to ensure the homogeneity of the samples (Supplementary Figure S1). The initial crystallization screening was carried out using Natrix (Hampton Research), which produced microcrystals under several conditions. After modifications of the crystallization conditions, we eventually obtained an elongated crystal (10 × 10 × 100 μm) with 50 mM MES monohydrate (pH 5.6), 20 mM KCl, 10 mM MgSO_4_ heptahydrate, 7% (v/v) PEG400 for the Nrf2–MafG–CsMBE1 and CsMBE2 complexes, and 50 mM Tris–HCl (pH 7.5), 100 mM KCl, 10% polyethylene glycol monomethyl ether 550, 2% (v/v) glycerol for the Nrf2(A510Y)-MafG-CsMBE2 complex. The crystals were stepwise transferred to buffers containing the crystallization condition with an increasing amount of glycerol (final 25%, increased by 5%) and then flush-cooled in a cold gas stream of nitrogen. The diffraction data were obtained at beamlines NE3A, BL5A and BL17A at the Photon Factory (Tsukuba). Indexing and integration were performed using HKL2000 (22). The structure was determined by molecular replacement with the MafG-DNA complex (PDB code 3A5T) as a search model using the Crystallography and NMR System (CNS version 3.0) (23). In all three structures, there are two Nrf2–MafG–DNA complexes in the asymmetric unit. Structural refinement was performed with Coot (24), CNS, and Phenix (25), without NCS restraints. The structural figures were made using the program PyMOL (https://www.pymol.org/).

### Sequence analysis

A multiple sequence alignment of 34 CNC family proteins containing homologs of Nrf2, Nrf1, NF-E2, BACH1 and BACH2 was created using Clustal W (26). The full alignment is available as Supplementary text S1. The sequence logo was created with the WebLogo server (27).

### Electrophoretic mobility shift assay (EMSA)

For the EMSA, a longer Nrf2 fragment (residues 182–560) and its mutants were used to distinguish bands corresponding to Nrf2–MafG–DNA and MafG homodimer-DNA. The synthesized 30-mer oligonucleotides (Supplementary Table S1) were annealed and purified using PAGE. The resulting 30 bp double-stranded fragment was labeled with [γ-32P] ATP using T4 polynucleotide kinase (TOYOBO).

To determine the KD values, the dimers were mixed with the 32P-end labeled DNA probe (at about 1.6 pM) in a gel shift buffer (20 mM Tris–HCl pH 7.2, 150 mM KCl, 10 mM DTT, 8 mg/ml bovine serum albumin, 0.005% Tween 20, and 50 ng/ml Poly(dI-dC), 2.5% Ficol), and incubated for 30 min (Jun–Fos heterodimer) or 16 h (Nrf2–MafG heterodimer and MafG homodimer). The Nrf2–MafG heterodimer and the MafG homodimer were incubated for a longer time to ensure equilibration, as Maf proteins are known to have slow binding kinetics (28). To confirm the formation of the Nrf2–MafG heterodimer, Nrf2 and MafG were mixed at a molar ratio of 3:1, and for calculation, the MafG concentration was assumed to be that of the heterodimer (29).

For the competitive EMSA assays, 1 nM of MafG (21–123) fragment was first mixed with the 32P-end labeled DNA probe (at about 1.6 pM) to produce the MafG dimer-DNA complex, against which the wild-type and mutant Nrf2 fragments (182–560) were titrated in a gel shift buffer. The resulting mixtures were incubated at 4°C for 16 h.

For both assays, the protein-DNA mixtures were electrophoresed with 8% non-denaturing polyacrylamide gel in 0.5× TBE buffer at 150 V and 4°C for 1 h. Radioactivity was visualized using autoradiography (Fujifilm BAS2500), and the autoradiographs were quantified in Multi Gauge V3.0 software (Fujifilm). Kinetic parameters and EC50 were derived by fitting the values to the Sigmoidal model using KaleidaGraph 4.5.3 software.

### Cell culture and reporter gene assay

The HEK293T cells were purchased from the Japanese Collection of Research Bioresources (National Institute of Health Sciences) and maintained in Dulbecco's modified Eagle's medium containing 1 g/l D-glucose, 0.58 g/l l-glutamine, and 0.11 g/l sodium pyruvate (Nacalai Tesque) with 10% fetal bovine serum and 1% penicillin-streptomycin at 37°C in 5% CO_2_ atmosphere. HEK293T cells (2.5 × 10^4^ cells/well) cultured in 96-well plates were transfected with 30 ng pCMV-Tag2B-Nrf2 plasmid (encoding Flag- and His6-tagged full-length Nrf2 wild type or its mutants), 1 ng pact-RL plasmid (30) (encoding the Renilla luciferase as an internal control), 40 ng pGL4.10 [luc2]-Nqo1 plasmid (encoding the firefly luciferase downstream of the Nqo1 enhancer containing one copy of CsMBE), and 30 ng empty pact1 plasmid (31) with Lipofectamine LTX reagent (Thermo Fisher Scientific). The optimal amount of pCMV-Tag2B-Nrf2 was determined by a titration experiment in which the total amount of added pCMV-Tag2B-Nrf2 and pact1 was kept constant (60 ng) (Supplementary Figure S2). Twenty-four hours after transfection, luciferase activity was measured with the Dual-Luciferase Reporter Assay System (Promega) according to the manufacturer's instructions with the use of a Centro LB960 microplate luminometer (Berthold). For each Nrf2 construct, the data were collected in quadruplicate. The data were normalized by the measurements of cells without pCMV-Tag2B-Nrf2 plasmid as a control in the same plate. Means and standard deviations were calculated with the normalized values from different plates.

### Molecular dynamics (MD) simulation

We performed canonical MD simulations for four molecular systems consisting of the Nrf2–MafG heterodimer complexed with a DNA fragment. Each system has a different base pair at the fourth position of the DNA chain, which is an adenine interacting with Asn507 via a water molecule in the crystal structure. The three other models with thymine, guanine, and cytosine for this position were modeled using 3DNA software (32) on the basis of the crystal structure. For all models, a base was added to the 3′-end of the DNA chain to complement the unmatched 5′-end of the complementary chain. The water molecule-mediating interactions between Asn507 and dA4 in the crystal structure were removed from the models. The purpose was to compare the interaction stability of the four kinds of DNA sequences. Because this water can work as an interaction mediator between Nrf2–MafG only for the wild-type sequence (with dA4), placing it in the initial configuration of the systems can benefit from stabilizing DNA recognition for the dA4 sequence but not for other sequences. To address this bias, water was removed from the system. For each model, the Nrf2–MafG–DNA complex was bathed in 150 mM NaCl explicit solvent in the periodic boundary cell, the dimension of which was ca. 140 Å. This cell size ensured that the margin between the edge of the solute and the cell boundary was more than 10 Å for all six directions. Before the production run of the MD simulations, the two methods of energy minimization, the steepest descent and the conjugate gradient, were applied successively. The systems were then relaxed via a 1.0 ns run under the NPT ensemble with position restraints for the heavy atoms. The temperatures of the systems were gradually heated from 10 K to 300 K during the first 0.5 ns of this run by applying the Berendsen thermostat with the Parrinello–Rahman barostat. Then, four independent NVT runs lasting over 300 ns with different randomly generated initial atomic velocities were performed. The total simulation length was 4.8 μs. The temperature was kept at 300 K with a Nosé–Hoover thermostat (33). The trajectory of the last 270 ns in each run was analyzed as a production run.

For all simulations, the AMBER ff99SB-ILDN force field (34) with bsc0 modification (35) was applied as a potential parameter for polypeptides and DNA. An explicit solvent was treated with the TIP3P water model (36) and the ion parameters proposed by Joung and Cheatham (37). Electrostatic potentials were calculated using the particle mesh Ewald method (38). The non-bonded pair potentials were cut off at 12 Å. The covalent bonds with hydrogen atoms were constrained using the LINCS method (39). The integration time step was 2.0 fs. All simulations were performed using Gromacs 5.0.4 (40). Hydrogen bonds observed in the simulation trajectories were analyzed using the MDAnalysis library (41) with a threshold of ≤3 Å for donor–acceptor distance and >120° for the donor–hydrogen–acceptor angle.

### Fragment molecular orbital (FMO) analysis

The pretreatment of the MafG-Nrf2 complex bound to CsMBE1 obtained by X-ray crystallography was executed using MOE software (42) to perform the FMO calculation. All the water molecules of the complex were removed. The missing heavy atoms of the side chain on the protein and the nucleic acid were structurally complemented by the *StructurePreparation* function and hydrogen atoms added by the *Protonate3D* function. Only the complementary heavy and hydrogen atoms were structurally optimized with the Amber10:EHT force field, and this structure was used as the MafG-Nrf2 complex (the CsMBE1 complex). In addition, to analyze the effect on the molecular recognition of the methyl group for the dT14* base, the MafG-Nrf2-DNA complex containing the C-G base pair at the position of the dA14-dT14* pair of the CsMBE1 DNA (the dC14* complex) was constructed based on the pretreated CsMBE1 complex structure. Only the substituted nucleic acid site was structurally optimized with the Amber10:EHT force field.

We briefly describe the *ab initio* FMO method (43). A large molecule or a molecular cluster is divided into small fragments, and MO calculations for each fragment monomer and dimer are performed to obtain the properties of the entire system. The many-body effects were considered through environmental electrostatic potentials. The total energies of the FMO calculations are given by

(1) $$\begin{equation*}{\rm{\;}}{E_{{\rm{total}}}} = \mathop \sum \limits_I E{^{\prime}_I}\; + \mathop \sum \limits_{I >J} \Delta {\tilde E_{IJ}},\end{equation*}$$

where *E'_I_* is monomer energy without the environmental electrostatic potential, Δ*E _IJ_* is the inter-fragment interaction energy (IFIE), and *I* and *J* are fragment indices. In addition, pair interaction energy decomposition (PIEDA) (44,45) was used to analyze the energy components of IFIE, $\Delta {\tilde E_{IJ}}$: electrostatic (ES), exchange (EX), charge transfer with others (CT + mix), and dispersion interaction (DI), where

(2) $$\begin{equation*}{\rm{\;}}\Delta {\tilde E_{IJ}} = \;\Delta \tilde E_{IJ}^{{\rm{ES}}} + \Delta \tilde E_{IJ}^{{\rm{EX}}} + \Delta \tilde E_{IJ}^{{\rm{CT}} + {\rm{mix}}} + \Delta \tilde E_{IJ}^{{\rm{DI}}}.\end{equation*}$$

The DI energy corresponds to the electron correlation energy estimated by second-order Møller–Plesset perturbation (MP2) theory.

All FMO calculations of the CsMBE1 and dC14* complexes were performed at the FMO2-MP2/6–31G* level with Cholesky decomposition approximation (46) using the ABINIT-MP program (47,48). The unit of fragment for proteins was each amino acid residue for the protein, and each base and backbone for DNA was treated as a single fragment. The interaction energy between the ligand and each amino acid residue was analyzed through IFIE analysis and PIEDA using Biostation Viewer (48). Atomic charge distribution with natural population analysis was performed at the HF/6–31G* level. The FMO calculation results of the CsMBE1 and dC14*complexes are registered in the FMO database (49,50).

## RESULTS

### Overall structure

**Figure 1.**
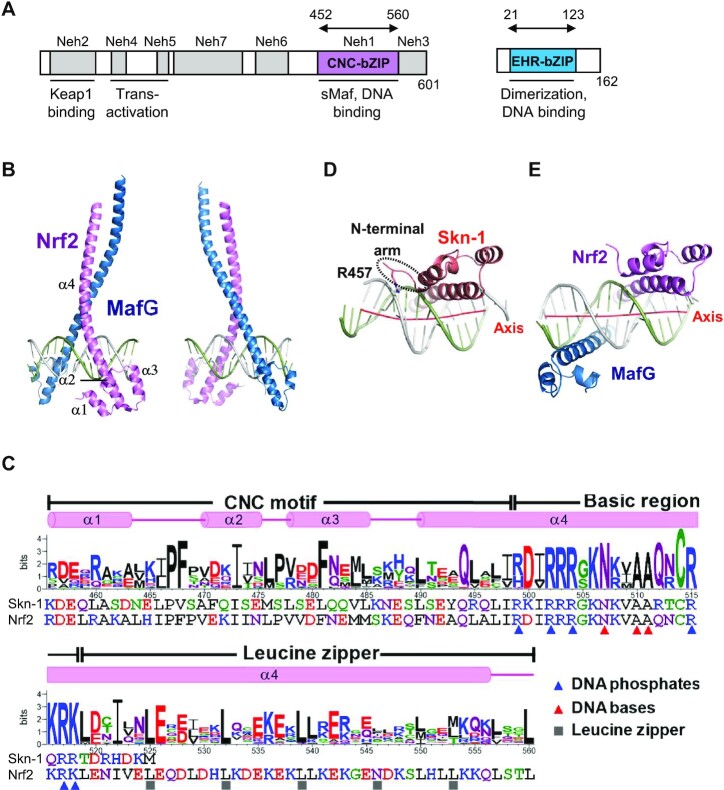
Structure of Nrf2–MafG–CsMBE1. (**A**) Conserved regions of Nrf2 and M + afG. Seven conserved regions of Nrf2 designated as Neh (Nrf2-ECH homology) 1–7 are shown along with th + eir functions. Arrows indicate the fragments used for the structural study. (**B**) Overall structure of the Nrf2–MafG–CsMBE1 complex. (**C**) Sequence logo created from 34 CNC family proteins, containing homologs of Nrf2, N+-rf1, NF-E2, BACH1 and BACH2. The sequences of human Nrf2 and *C. elegans* Skn-1 and the Nrf2 secondary structural elements are also shown. The residues interacting with DNA phosphates and bases in the Nrf2–MafG–CsMBE1 complex are indicated by blue and red triangles, respectively. Residues that mediate intermolecular interactions (mainly hydrophobic) at the **d** positions of the heptad repeats in the leucine zipper region are indicated by gray cubes. (D, E) Bottom views of the Skn-1 DNA-binding domain in a complex with DNA (PDB ID 1SKN) (**D**) and the Nrf2–MafG–CsMBE1 complex (**E**). The red lines show the helical axes of the bound DNA double helices in each complex.

**Figure 2.**
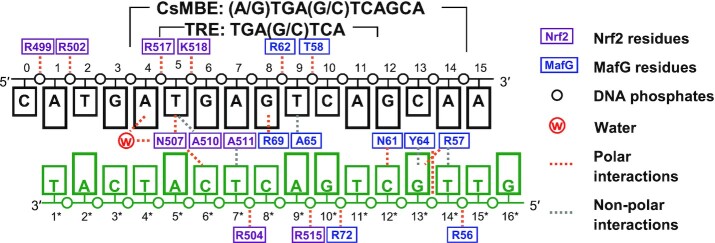
Schematic diagram of protein–DNA interactions in the Nrf2–MafG–CsMBE1 complex. Polar and non-polar interactions are indicated by orange and gray dashed lines, respectively.

We solved the crystal structures of the heterodimer between the DNA-binding domains of human Nrf2 (residues 452–560) and MafG (residues 21–123) (Figure 1A) in complexes with two kinds of DNA fragments containing CsMBE (CsMBE1: 5′-ATGATGAGTCAGCAA-3′ and CsMBE2: 5′-ACAGTGACTCAGCAG-3′, where CsMBE elements are underlined) at 2.3 and 2.6 Å resolutions, respectively (Figures 1B, C, and 2; Supplementary Figures S3 and S4; and Supplementary Tables S1 and S2). As the CsMBE1-containing complex was solved at a higher resolution, we mainly discuss this complex here, unless otherwise noted.

The overall view of the Nrf2–MafG–DNA complex is shown in Figure 1B, and a schematic diagram of the protein–DNA interactions is presented in Figure 2. The DNA-binding domain of Nrf2 contains the N-terminal three short α-helices (α1–α3), followed by a large one (α4). The CNC motif (residues 456–498) constitutes the first three helices and a part of α4. Furthermore, α4 contains the basic region (residues 499–518) and the leucine zipper (residues 519–560), which mediate the interactions with DNA and MafG, respectively (Figure 1C). The DNA-binding domain of MafG contains the N-terminal EHR, followed by the basic and leucine zipper regions. Like other bZIP proteins, Nrf2 and MafG each contact a half site of the CsMBE. Three Nrf2 residues (Asn507, Ala510, and Ala511) recognized the central TRE half site (positions 5–7, 5′-TGA-3′) in a manner similar to that of AP-1 proteins (Figure 1C and Supplementary Figures S4, S5).

The solution structure of the isolated CNC-bacic region of Nrf2 (residues 445–523) was determined by NMR spectroscopy (PDB 2LZ1). In the absence of DNA and sMaf proteins, a part of the basic region of Nrf2 (residues 499–504) forms a helix in the four-helix bundle structure with the CNC motif, whereas resides 505–523 are flexible (Supplementary Figure S6A). Structural superposition revealed that the region folded in the NMR structure does not undergo significant conformational changes upon binding to DNA (Supplementary Figure S6B). These observations suggest that the N-terminal side of the Nrf2 basic region adopts a preformed DNA-recognition helix stabilized via interactions with the CNC motif, while its C-terminal part becomes folded upon binding with sMaf and DNA. This feature is reminiscent of MafG, whose basic region and EHR motif constitute the preformed DNA-binding unit in the absence of DNA (51), while many other bZIP proteins have just basic regions that are rather flexible in the free state and adopt the helical structure only upon binding to DNA (52).

### Distinct DNA-binding modes of Nrf2 and Skn-1

Skn-1 is a transcriptional factor regulating stress responses in *C. elegans* and is thought to be an evolutionary-related functional homolog of Nrf2 with a limited sequence homology (1,53,54). It has the CNC motif and the basic region, but it does not contain the leucine zipper region and therefore binds DNA as a monomer. It also has a unique stretch of conserved basic residues located at the N-terminal to the CNC motif (N-terminal arm), which contacts the DNA phosphates and thus contributes to monomeric high-affinity binding to the target DNA.

To understand how the difference in the DNA binding modes of Skn-1 and CNC family proteins is related to their transcriptional regulation functions, we compared the structures of the current Nrf2–MafG–DNA complex with that of the monomeric Skn-1 bound with DNA (55). In the Skn-1 complex, the bound DNA is significantly bent because of the interaction between Arg457 in the N-terminal arm and one phosphate group of DNA (Figure 1D). On the other hand, the overall conformation of the DNA in the Nrf2–MafG complex is almost straight, reflecting pseudosymmetrical interactions between the two bZIP proteins and DNA (Figure 1E). This straight DNA conformation is incompatible with the interactions via the N-terminal arm, as seen in the Skn-1 complex. The distinct DNA-binding modes suggest the divergent transcriptional regulatory mechanisms employed by these factors. Unlike monomeric Skn-1, Nrf2 lacks the N-terminal arm and thus does not have enough affinity to bind DNA as a monomer. Instead, the tailless Nrf2 does not induce DNA bending and thus binds DNA as heterodimers with sMaf proteins by forming pseudosymmetrical interactions. The heterodimer formation in higher eukaryotes allows for fine-tuned transcriptional control, depending on the relative and absolute abundance of the components.

### Structural comparison of the Nrf2–MafG–DNA and the MafG homodimer-DNA complexes

**Figure 3.**
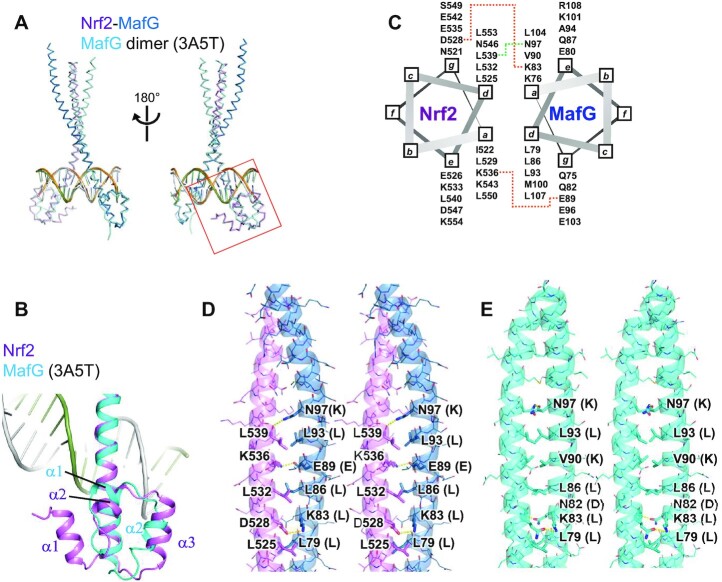
Structural comparison of the Nrf2–MafG–DNA and the MafG homodimer-DNA complexes. (**A**) Structural superposition of the Nrf2–MafG–DNA complex and the MafG homodimer-DNA complex (PDB ID: 3A5T). The region shown in Figure 3B is indicated by a red box. (**B**) Structural comparison of the CNC motif of Nrf2 and the EHR motif of MafG. (**C**) A helical wheel diagram showing intermolecular interactions. The orange and green dotted lines indicate intermolecular salt brides and a hydrogen bond, respectively. (D, E) Stereo views of the dimerization interfaces of (**D**) the Nrf2–MafG heterodimer and (**E**) the MafG homodimer. Residues that mediate intermolecular interactions are shown in the stick model. The characters in parentheses next to the MafG residue labels indicate the corresponding amino acids of Nrf2.

Figure 3A shows the structural superposition of the Nrf2–MafG heterodimer with the MafG homodimer bound with the MARE DNA. The basic regions of the two proteins in the two complexes superpose well (a root mean square deviation of 0.53 Å for 40 Cα atoms in the basic regions of the two proteins), showing that the two dimers bind DNA similarly. Compared to the EHR motif of MafG, the CNC motif of Nrf2 contains an additional helix at the N-terminus (α1), which forms extensive interactions with the other region of the CNC motifs (see below). The following two helices (α2) and (α3) correspond to the α1 and α2 helices of HER, but their locations and directions are slightly different (Figure 3B). The MafG subunit of the two complexes recognizes the MARE half site essentially the same way (Figure 2), mainly contacting the outer base pairs (dG12–dC13–dA14) but not the inner bases constituting the TRE motif (13).

The leucine zipper motifs of Nrf2 and MafG dimerize through conserved hydrophobic residues (mainly Leu) located at the **d** positions of the heptad repeats (**a**–**g**) (Figures 1C, 3C and D). In addition, there are two intermolecular salt bridges mediated by side chains (Nrf2 Asp528-MafG Lys83 and Nrf2 Lys536-MafG Glu89) and one hydrogen bond between the main chain oxygen of Nrf2 Leu539 and the side chain of MafG Asn97 (Figure 3C, D). Asp528 and Lys536 of Nrf2 are relatively conserved among CNC family proteins (Figure 1C), suggesting that these interactions with sMaf proteins are shared in the CNC family. A comparison of the intermolecular hydrophilic interactions in the Nrf2–MafG heterodimer and the MafG homodimer (Figure 3E) shows that Lys83 and Asn97 of MafG are involved in both complexes. Lys83 and Asn97 of MafG are not shared by Nrf2 (Figure 3D and E), which may explain why Nrf2 preferentially forms heterodimers with sMaf proteins rather than homodimers.

### Structure of the CNC motif

**Figure 4.**
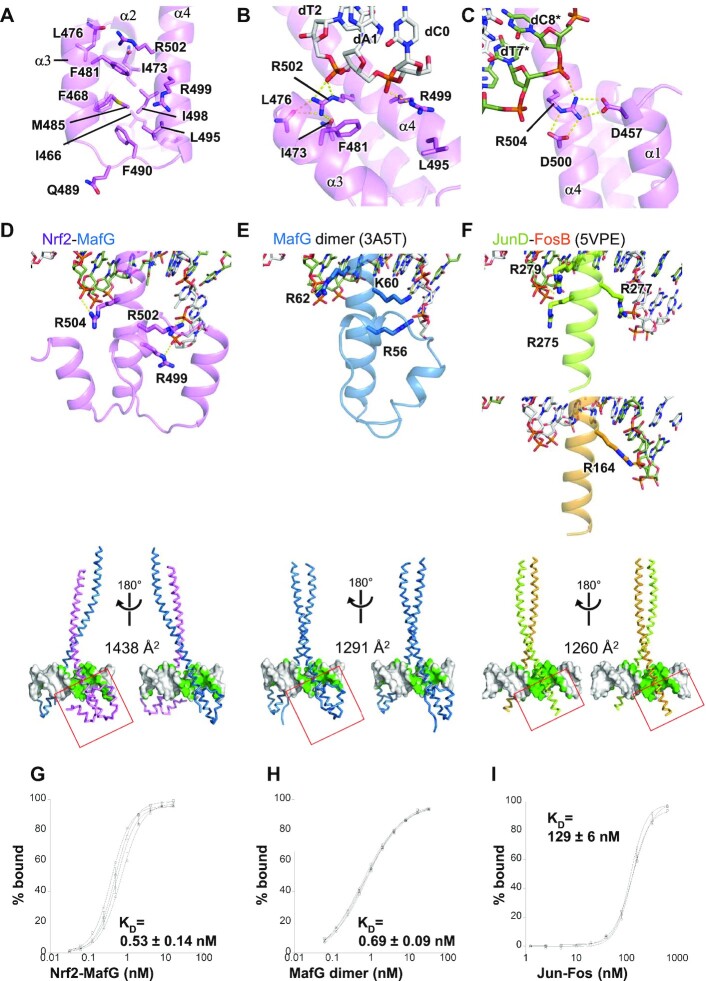
Structure of the CNC motif and comparison with other bZIP proteins. Hydrophilic interactions are indicated by yellow dashed lines. (**A**) Hydrophobic residues of the CNC motif. (B, C) Binding of DNA phosphates by Arg499 and Arg502 (**B**) and by Arg504 (**C**) of Nrf2. (D–F) Top, the DNA interactions of the N-terminal part of the basic regions of the indicated bZIP proteins. The regions shown in the bottom panels are indicated by red boxes. Bottom, DNA contact surfaces in green color. (**D**) Nrf2–MafG heterodimer-DNA, (**E**) MafG homodimer-DNA (PDB ID 3A5T), and (**F**) JunD–FosB heterodimer-DNA (PDB ID 5VPE). (G–I) Titration curves of (**G**) the Nrf2–MafG heterodimer, (**H**) MafG homodimer, and (**I**) Jun–Fos heterodimer toward DNA containing CsMBE, as analyzed by the EMSA. The results from four (Nrf2–MafG) or three (MafG homodimer and Jun–Fos) independent experiments are shown with their standard deviations. The calculated *K*_D_ values are also shown.

Figure 4A–C shows close-up views of the CNC motif and its role in DNA binding. The four helices of the CNC motif pack against one another, and they interact extensively with the N-terminal portion of the basic region. The conserved hydrophobic residues (including Ile466, Phe468, Phe481, Met485, Phe490, Leu495 and Ile498) form a hydrophobic core and help in the formation of the continuous helical structure of α4 spanning residues 490–556 (Figures 1B and 4A). As a result, the conformation of the three Arg residues in the basic region (Arg499, Arg502 and Arg504), which interacts with the DNA phosphates, is stabilized at specific positions. Arg499 forms a van der Waals interaction with Leu495 and a salt bridge with the dA1 phosphate of the top strand (Figures 2 and 4B; the DNA bases of the top and bottom strands are indicated without and with *, respectively, and numbered from the 5′ to 3′ direction in the top strand so that the base-pairing residues have the same residue numbers). Arg502 stacks against Phe481, forms hydrogen bonds with the main chain carbonyl groups of Ile473 and Leu476, and forms a salt bridge with the dT2 phosphate (Figures 2 and 4B). On the other side of the CNC motif, Asp457 and Asp500 form salt bridges with Arg504, which, in turn, interacts with the dT7* phosphate (Figures 2 and 4). Thus, the CNC motif contributes to the DNA phosphate recognition of Nrf2 through the conformational restriction of these arginine side chains.

We then compared the DNA-binding modes of the Nrf2–MafG heterodimer and other bZIP proteins. The N-terminal side of the basic region of Nrf2 has three basic residues that contact DNA phosphates (Arg499, Arg502 and Arg504) (Figure 4D). MafG also has three basic residues that could contact DNA phosphates (Arg56, Lys60, and Arg62) in the N-terminal side of the basic region (Figure 4E), while in the Nrf2–MafG heterodimer and one MafG protomer in the MafG heterodimer Lys60 does not bind DNA (Figure 2). Thus, the Nrf2–MafG heterodimer and the MafG homodimer both have five basic residues contacting the DNA phosphates in the N-terminal side of the basic region. On the other hand, canonical bZIP proteins without CNC or EHR motif have fewer basic residues contacting the DNA phosphates at the corresponding regions; there are four, two, and two residues in the JunD–FosB heterodimer (56), CREB1 homodimer (57), and C/EBPβ homodimer (30) complexes, respectively (Figure 4F and Supplementary Figure S7). Notably, Arg499 of Nrf2 contacts the DNA phosphates up to four nucleotides upstream of the core TRE segment (Figure 2), while no other bZIP protein binds the DNA phosphate at the same position. Many bZIP proteins have lysine at the position corresponding to Nrf2 Arg499 (Supplementary Figure S8); however, these lysine residues point away from the phosphate, are disordered, or are not included in the crystallization constructs. Consequently, the Nrf2–MafG heterodimer forms more extensive interactions with DNA compared with canonical bZIP dimers (Figure 2). The DNA surface area buried by the Nrf2–MafG heterodimer is 1438 Å^2^ (Figure 4D), which is comparable to those seen in Maf homodimers (1291–1412 Å^2^; Figure 4E and Supplementary Figure S7C, D) (28,58) and larger than canonical bZIP complexes (1097–1260 Å^2^) (Figure 4F and Supplementary Figure S7A, B).

### CNC motif is essential for high-affinity DNA binding and activator functions

The larger buried surface, due to extensive interactions with DNA, suggests that the Nrf2–MafG heterodimer binds DNA more tightly than other bZIP proteins. To test this directly, we measured the binding affinities of the Nrf2–MafG heterodimer and the MafG homodimer for a DNA fragment containing CsMBE (and thus the internal TRE element) and compared them with that of the Jun–Fos heterodimer for the same DNA fragment using the EMSA (Spplementary Table S1 and Supplementary Figures S9, and S10). We found that the Nrf2–MafG heterodimer binds to the CsMBE with a *K*_D_ value of 0.53 nM (Figure 4G), which agrees with our previous measurements based on the surface plasmon resonance assay (59). The MafG homodimer binds the same DNA fragment with a comparable K_D_ value (0.69 nM, Figure 4H). Interestingly, under the same experimental condition, the Jun–Fos heterodimer bound to the same DNA fragment approximately 200 times more weakly (*K*_D_ value: 129 nM, Figure 4I), even though it contained the canonical TRE element.

**Figure 5.**
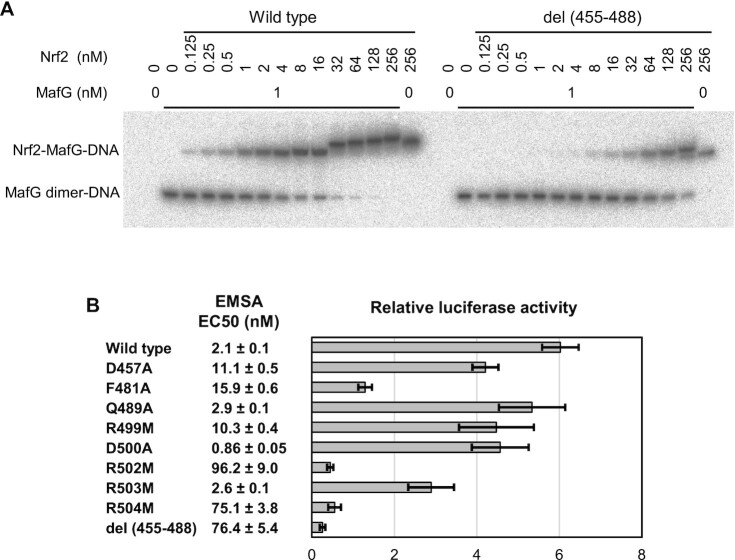
Mutational analysis. (**A**) Representative gel image of the competitive EMSA using a fixed concentration (1 nM) of MafG and an increasing concentration (0.125 to 256 nM) of wild-type Nrf2 and the del (455–488) mutant. The full gel images of the competitive EMSA are shown in Supplementary Figure S11. (**B**) Summary of the competitive EMSA and luciferase reporter assay. EC50, the effective Nrf2 concentration at which 50% of the DNA is bound with the Nrf2–MafG heterodimer, was calculated based on 13 (wild type) or three (mutants) EMSA experiments. Samples with no exogenous Nrf2 expression were used as the control in the luciferase reporter assay.

We then performed a competitive EMSA to analyze how the Nrf2–MafG heterodimer and the MafG homodimer competitively bind to the CsMBE element (Figure 5A and Supplementary Figures S11 and S12). In this assay, titration of the DNA with Nrf2 was performed under a fixed concentration (1 nM) of MafG. As the concentration of Nrf2 increases, the signal of the Nrf2–MafG heterodimer-DNA complex becomes stronger, whereas those of the MafG homodimer-DNA complex and free DNA become weaker. To quantify the binding affinity, we calculated the effective concentration at which 50% of the DNA was bound with the Nrf2–MafG heterodimer (EC50). The wild-type Nrf2 was shown to have an EC50 value of 2.1 nM (Figure 5B and Supplementary Figures S11 and S12). At the Nrf2 concentration of 16 nM or more, the mobility of the shifted band gradually decreased, suggesting that excess Nrf2 molecules may bind to the DNA in a non-specific manner. Even without MafG, a shifted band was observed when the highest concentration of Nrf2 (256 nM) was used (Figure 5A and Supplementary Figure S11). These results suggest that, contrary to previous reports, at higher concentrations, Nrf2 alone might bind regulatory DNA elements (possibly as homodimers) and activate transcription in cells. Further studies are needed to determine whether this non-canonical binding may have physiological or pathological roles under certain circumstances, such as in some cancers where the cellular Nrf2 level is aberrantly high.

We then created a series of Nrf2 mutants and tested their DNA-binding activity using the competitive EMSA (Figure 5B and Supplementary Figures S9, S11, and S12). We first examined the roles of three arginine residues (Arg499, Arg502 and Arg504) that contact the DNA phosphates, as well as that of Arg503, which does not contact DNA phosphates in the CsMBE1 complex (Figures 2 and 4D). To remove the positive charge while maintaining a long side chain, we individually substituted them with methionine. The results of the competitive EMSA showed that the R502M and R504M mutants had a significantly reduced DNA affinity (more than a 30-fold reduction based on their EC50 values), whereas the R499M mutant exhibited a smaller reduction of an affinity (about fivefold), and R503M retained an affinity comparable to that of the wild type. These results demonstrated the important roles of Arg499, Arg502 and Arg504 for high-affinity CsMBE binding by the Nrf2–MafG heterodimer.

We next tested the del (455–488) mutant, in which helices α1–α3 were deleted (Figure 4A), to analyze the role of these helices of the CNC motif. The del (455–488) mutant exhibited strikingly weaker DNA binding, comparable to those of the R502M and R504M mutants. This result shows that although the three helices do not form direct hydrophilic interactions with DNA, they contribute to the high DNA affinity of the Nrf2–MafG heterodimer, possibly by stabilizing the key DNA-binding residues in the basic region at the positions suitable for DNA binding. To test this hypothesis, we individually introduced alanine substitution at Asp457 (on α1) and Phe481 (on α3), which stabilize the conformations of the DNA-contacting Arg504 and Arg502 (Figure 4B and C). The D457A and F481A mutants exhibited about fivefold and sevenfold reductions in DNA affinity, respectively, demonstrating that these residues indeed contribute to the high DNA affinity. As a control, alanine substitution at non-conserved Gln489 (on the α3–α4 loop in the CNC motif), located away from the DNA-binding residues (Figure 4A), did not significantly affect DNA affinity. We also examined the effect of alanine substitution at Asp500, another residue that interacts with Arg504 (Figure 4C). Contrary to our prediction, the D500A mutant exhibited a small but detectable increase in DNA affinity (about twofold).

Having identified some of the residues that are important for high-affinity DNA binding by the Nrf2–MafG heterodimer, we next conducted a luciferase reporter assay (60) to analyze the transcription activation activity of the same set of mutants used in the EMSA (Figure 5B and Supplementary Figure S2). The R502M, R504M, and del (455–488) mutants exhibited transcriptional activities that were even weaker than those of the control sample in which no Nrf2 was exogenously expressed, suggesting a dominant-negative effect of these mutants. F481A exhibited significantly reduced activity comparable to that of the control. D457A, R499M, D500A, and R503M exhibited weaker but still considerable (50%–70% of the wild type) activities, whereas Q489A was as active as the wild type. Except for D500A and R503M, the mutants examined exhibited consistent DNA affinity and transcriptional activity. Taken together, these results demonstrate that Asp457 and Phe481 in the CNC motif and three phosphate-contacting arginine residues (Arg499, Arg502 and Arg504) are important for the high-affinity DNA binding and transcriptional activation by the Nrf2-sMaf heterodimer.

### Water-mediated recognition of purine at the flanking position

**Figure 6.**
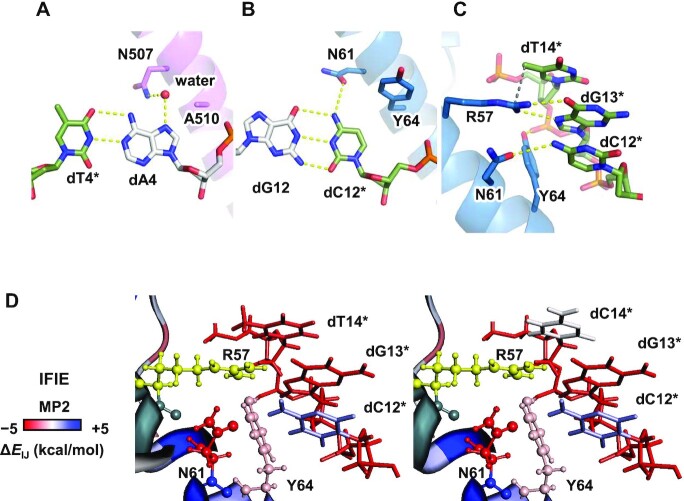
Recognition mechanisms of the bases outside the core TRE element. (**A**) Water-mediated recognition of dA4 by Asn507 of Nrf2. (**B**) Recognition of dC12* by Asn61 and Tyr64 of MafG. (**C**) Recognition of the 5-methy group of dT14* by Arg57 of MafG. The 5-methyl carbon of dT14* and the guanidium nitrogen of Arg57 of MafG are connected with a gray dashed line. (**D**) IFIE analysis. The residues near Arg57 are colored according to their interaction energy with Arg57 (red: favorable, white: neutral, and blue: unfavorable). Arg57 is colored yellow.

ChIP-seq studies have revealed that the CsMBE consensus sequence contains a conserved purine base next to the core TRE element (dA4 of CsMBE1 in the present study) (61,62). Previous results have consistently shown that the affinity of the Nrf2–MafG heterodimer toward DNA is decreased by approximately twofold when the purine base at this position is replaced with a pyrimidine base (59). In the crystal structure, the N7 atom of dA4, which is unique to purine bases, is recognized by Nrf2 Asn507 via water-mediated hydrogen bonds (Figure 6A). The bridging water molecule is clearly visible in the electron density map of the CsMBE1 complex structure (Supplementary Figure S3).

We addressed whether the water-mediated interaction between the Asn residue and the purine base at the flanking position is seen in other bZIP proteins. Although the consensus sequence of the TRE has usually been referred to as 5′-TGA(C/G)TCA-3′, recent ChIP-seq and SELEX studies have revealed that the Jun–Fos dimer also preferentially binds to sequences containing purines at the 5′ side of the core TRE element (63,64). In the high-resolution crystal structures of Jun–Fos–DNA complexes (56), the conserved asparagine residues corresponding to Asn507 of Nrf2 (Asn165 of FosB and Asn278 of Jun-D) also form water-mediated hydrogen bonds with the N7 atom of the purine located at the 5′ location of the classical TRE sequence (Supplementary Figure S13). Thus, water-mediated purine recognition by conserved Asn residues is likely to be a common feature of CNC family and AP-1 proteins.

### Dynamics of water-mediated purine recognition

To analyze the dynamics of the interaction between the Nrf2–MafG heterodimer and DNA, we performed an MD simulation (Supplementary Figures S14, S15, and S16; Supplementary Table S3; and Supplementary Movies S1–S3). In particular, to examine the role of the water-mediated interaction between dA4 and Asn507, we created three model structures in which the base pair at the flanking position (dA4-dT4* in the CsMBE1 complex structure) is replaced with dG-dC, dT-dA or dC-dG, and then we conducted four 300-ns MD simulation runs for each of the four systems (the original complex and three base-replaced models). To remove potential bias, we deleted the coordinates of the bridging water from the structures. As shown in Supplementary Table S4, only in the complexes with a purine (dA4 or dG4) at the flanking position was water-mediated interaction predominantly (during 30% or more of the simulation period) formed between Asn507 and the base moiety, but no such interaction was stably found in the thymine and cytosine complexes. Taken together, our MD simulation shows that these interactions are not crystallization artifacts, and it supports the idea that Asn507 recognizes a purine at the flanking position via water-mediated hydrogen bonds with the N7 atom, thus contributing to the purine preference at this position by Nrf2-sMaf heterodimers and possibly other bZIP proteins. We also confirmed that the three Arg residues of Nrf2 (Arg499, Arg502, and Arg504) maintained their interactions with the DNA phosphates (Supplementary Figure S16). The intramolecular interactions between these Arg residues and the nearby residues were also stably maintained (Supplementary Figure S16). The intermolecular hydrogen bonds between the leucine zipper regions of Nrf2 and MafG were stable during the calculation (Supplementary Figure S16).

### Recognition of the MARE half site by MafG

The sMaf proteins share a characteristic Tyr residue in the basic region (Tyr64 of MafG), while CNC family and AP-1 proteins have Ala (Ala510 of human Nrf2) at the corresponding position. Interestingly, when this Ala residue of mouse Nrf2 (Ala502) was replaced with Tyr, the resulting mouse Nrf2(A502Y)-sMaf heterodimer preferentially bound MARE-like sequences with dG-dC dinucleotides located on both sides of the TRE core *in vitro* (59) and *in vivo* (3). These results suggest that the identity of the amino acid at this position is a critical determinant of the differential sequence specificities between CNC family and sMaf proteins.

To gain insight into such specificity switching by the Ala/Tyr replacement, we solved the 2.3-Å crystal structure of the complex containing CsMBE2, MafG, and the Nrf2 A510Y mutant (Supplementary Table S2 and Supplementary Figure S4) and compared the recognition mechanisms of the base pairs located on both sides of the TRE core (dA4-dT4* and dG12-dC12*) by wild type and A510Y Nrf2, as well as that by MafG. As noted above, the N7 atom of dA4 is recognized by Nrf2 Asn507 via water-mediated hydrogen bonds (Figure 6A). On the other hand, dC12* is directly hydrogen bonded with MafG Asn61 (corresponding to Nrf2 Asn507), accounting for the dC specificity of the sMaf proteins (Figure 6B). The bulky side chain of Tyr64 is located close to dC12*. Thus, if dC12* were replaced with purine, there would be no space for a water molecule to mediate the hydrogen bonds between Asn61 and the purine N7 atom. In fact, in the crystal structure of the human Nrf2(A510Y)-MafG heterodimer bound with CsMBE2 (containing dG4 next to the TRE core), no water molecule is observed bridging the dG4 N7 atom and Nrf2 Asn507 (Supplementary Figure S17A, B). Thus, the bulky Tyr at this position of Nrf2(A510Y) and possibly its equivalents in sMaf proteins are incompatible with water-mediated purine recognition by Nrf2 Asn507.

A SNP (rs242561) strongly correlated with the risk for Parkinsonian disorders was found in the Nrf2-sMaf binding site of the *MAPT* gene, which encodes the Tau protein (65). Compared with the low-risk T allele, the high-risk C allele is associated with a lower binding level of Nrf2 and the transcript level of the *MAPT* gene. The SNP position is located at the distal end of the half-MARE element (dT14*, corresponding to the low-risk allele). Nrf2-sMaf proteins prefer thymine at this position in vitro (29) and in vivo (61), for which no structural explanation has been provided. In the present structure, there is no hydrogen bond formed between the dA14-dT14* base pair and the Nrf2–MafG heterodimer, whereas the 5-methyl group of dT14* is located 3.5 Å away from the guanidium nitrogen of MafG Arg57, which recognizes dG13* via bipartite hydrogen bonds (Figure 6C).

The FMO method (43) can efficiently perform the quantum chemical calculation of large systems, such as biological macromolecules, by dividing them into small fragments, and it also provides an IFIE estimate. To elucidate the possible role of Arg57 in the recognition of dT14*, we conducted an FMO analysis of the current crystal structure (containing dA14-dT14*) and the modeled structure representing the high-risk allele, in which dA14-dT14* was replaced with dG-dC (Figure 6D). The results clearly demonstrated a favorable interaction between the Arg57 side chain and the base moiety of dT14*, which was lost when the base pair was replaced with dG-dC. The energy decomposition analysis (Supplementary Table S5) revealed that the interaction between Arg57 and dT14* is mainly governed by two terms: dispersion energy (possibly the CH-π interaction between the 5-methyl group of dT14* and the Arg57 guanidinium group) and electrostatic energy (possibly the Coulomb interaction between the partial negative charges at the 4-carbonyl group of dT14* and the positive charge at the guanidinium group of Arg57) (Supplementary Figure S18). Thus, our analyses explain a weak sequence preference for the dA-dT base pair of the half-MARE element by Nrf2-sMaf heterodimers, which underlies varying risks for Parkinsonian disorders associated with SNP rs242561.

## DISCUSSION

Our structural, functional, and computational analyses revealed the unique properties of CNC family and sMaf proteins. First, thanks to their CNC motif and perhaps the EHR, both CNC family and sMaf proteins exhibit DNA affinities that are much higher than those of other canonical bZIP family members, such as AP-1 proteins. Second, CNC family proteins have essentially similar sequence specificities as AP-1 proteins, whereas sMaf proteins recognize a more extended segment of DNA bases. Consequently, the Nrf2-sMaf heterodimer and the sMaf homodimer exhibit distinct, albeit similar, sequence preferences, consistent with our previous functional report (59).

**Figure 7.**
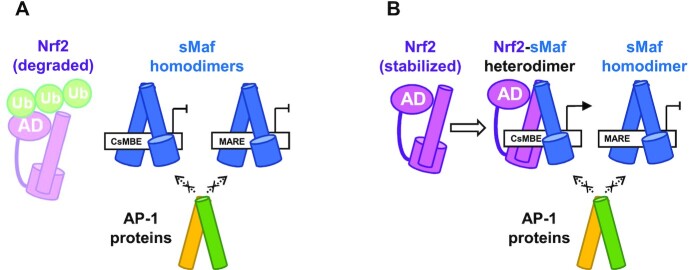
A model of precise transcriptional regulation by CNC and sMaf proteins. AD, activation domain. Ub, ubiquitin.

The binding of TFs to regulatory sites is influenced by multiple parameters, such as sequence preference, affinity, TF abundance, and cooperativity or competition between different TFs. The Nrf2–MafG heterodimer and the MafG homodimer are known to bind competitively to the same regulatory element containing CsMBE, thus enabling a precise and timely transcriptional regulation switch of their target genes in a manner dependent on the relative nuclear abundance of Nrf2 and MafG proteins. However, why ubiquitously expressed AP-1 proteins do not interfere with this regulation by aberrantly inducing these CsMBE-regulated genes although the TRE element is embedded in the CsMBE element remains elusive. Here, we propose that the high-affinity binding of the CNC family and sMaf proteins to their target sequences is essential for this regulatory mechanism (Figure 7). As sMaf proteins are constitutively and ubiquitously expressed, genomic CsMBE elements may usually be occupied by sMaf homodimers and are therefore repressed in most cells under normal conditions. Even when AP-1 proteins are induced by various stimuli, they may not bind to the CsMBE elements already occupied by the sMaf dimers because the DNA affinities of AP-1 proteins are remarkably low compared with those of the sMaf dimers. Once oxidative stress conditions occur, stabilized Nrf2 forms heterodimers with sMaf proteins, which can competitively bind to the CsMBE elements because the target DNA affinities of the Nrf2-sMaf heterodimer are comparable to those of the sMaf dimer. Thus, the Nrf2-sMaf heterodimer drives the transcriptional activation of the stress-response genes in a manner dependent on the sequences of the regulatory DNA elements and the relative abundance of Nrf2 and sMaf proteins, without being perturbed by other bZIP proteins, such as AP-1 proteins. The Nrf2–MafG heterodimer may less preferentially bind to the MARE elements, as the DNA affinity of the Nrf2–MafG heterodimer for the MARE elements is weaker than that of the sMaf homodimer.

Accumulating results show that other members of the CNC family also work in competitive ways (2). BACH1 and BACH2 are transcriptional repressors of the CNC family. BACH1 binds DNA under low heme concentrations, and the competitive binding of the BACH1–sMaf and Nrf2–sMaf heterodimers for the same regulatory element controls the expression of the heme oxygenase-1 gene in a heme level-dependent manner (9,10). In naïve CD8 + cells, BACH2 represses the genes activated by T cell antigen receptor signals by occupying their enhancers and thereby blocking DNA binding via AP-1 proteins (11). We assume that these BACH proteins also take advantage of their high-affinity DNA binding for the precise and timely regulation of their target genes.

## DATA AVAILABILITY

The atomic coordinates and structure factors for the Nrf2–MafG–CsMBE1, Nrf2–MafG–CsMBE2 and Nrf2 (A510Y)–MafG–CsMBE2 complexes have been deposited into the Protein Data Bank under accession codes 7X5E, 7X5F and 7X5G, respectively. The FMO calculation results of the CsMBE1 and dC14*complexes are registered in the FMO database (49,50) with FMODB IDs GN5R1 and 14NYZ, respectively.

## Supplementary Material

gkac1102_Supplemental_Files
