# Supplementary material for: Structural basis of transcription regulation by CNC family transcription factor, Nrf2
Source: Nucleic Acids Res. 2022 Dec 1;50(21):12543–57. doi: 10.1093/nar/gkac1102 (PMC9756947; doi:10.1093/nar/gkac1102)
Supplement: gkac1102_Supplemental_Files [file gkac1102_supplemental_files.zip › 221024SuppFigsTables.pdf]

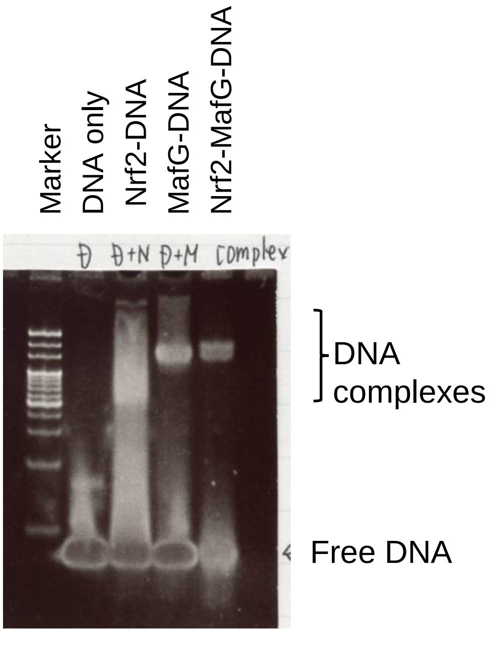

Supplementary Figure S1. Non-denaturing gel electrophoresis to monitor the formation of the Nrf2-MafG-DNA complex. Left, formation of DNA complexes. The heterodimer lane had a strong signal of free DNA, suggesting that more proteins were required. Right, the Nrf2-MafG-DNA complex under an optimized condition. Free DNA signal is weak, while the DNA complex band is sharp, suggesting the formation of the homogenous Nrf2-MafG-DNA complex.

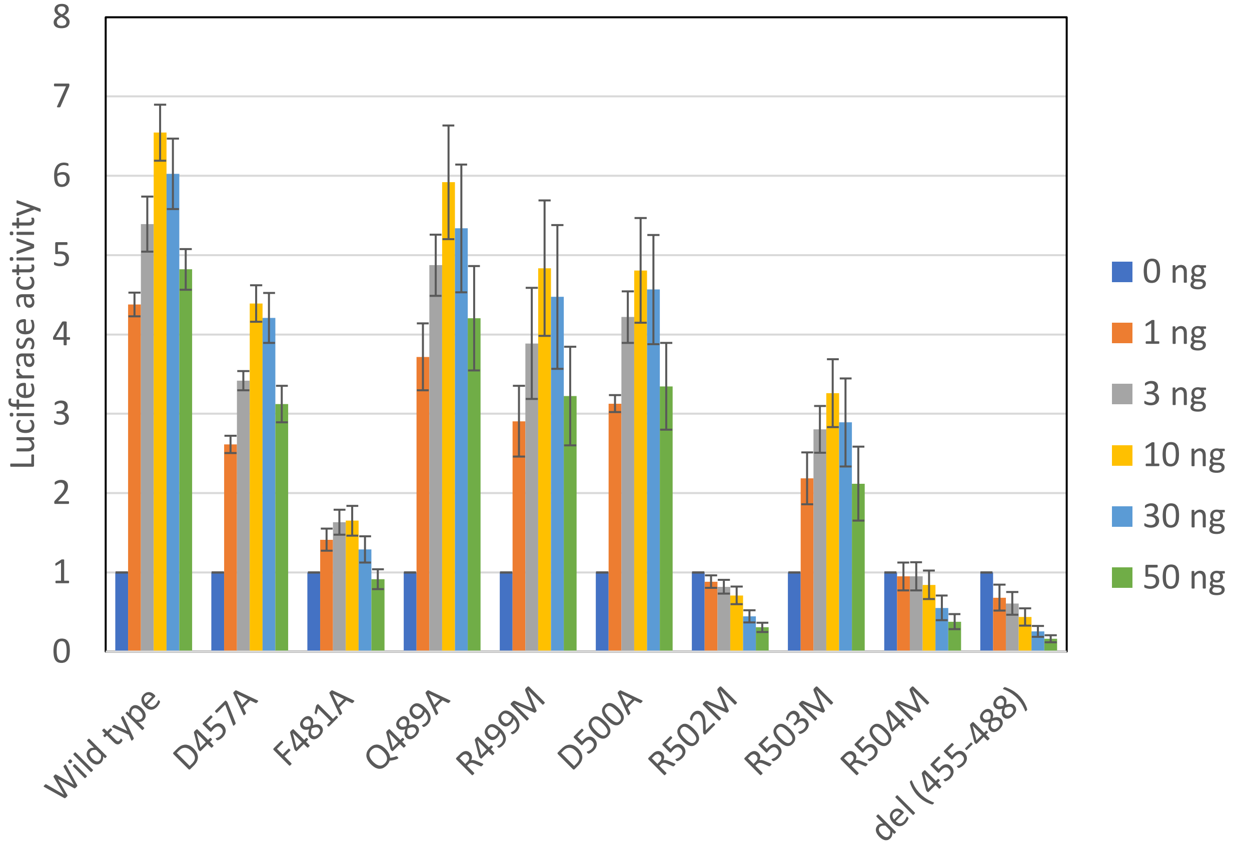

Supplementary Figure S2. A titration experiment to determine the optimal amount of Nrf2 vector for the luciferase assay.

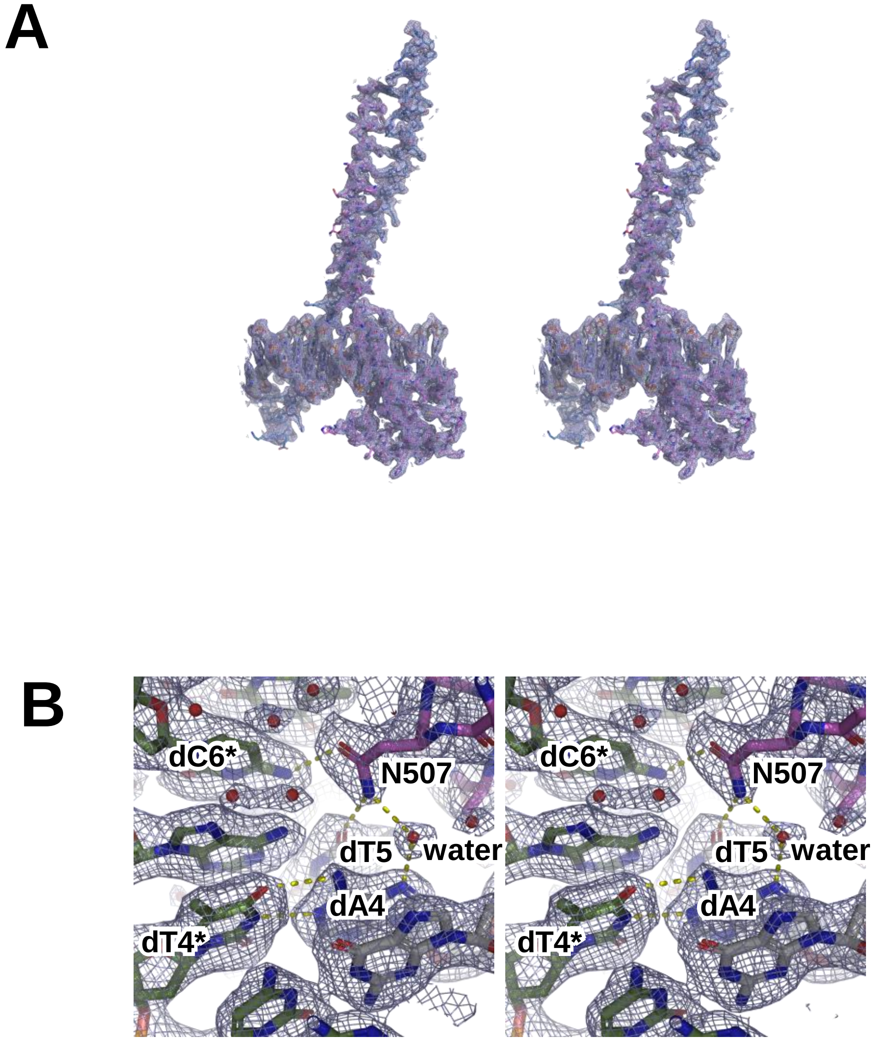

Supplementary Figure S3. Electron density maps (stereo views). (**A**), Overall structure of the Nrf2-MafG-CsMBE1 complex (shown as ribbon representation) and the 2mFo-DFc electron density map (blue, 1 σ). (**B**) Close-up view of the 2mFo-DFc electron density map (blue, 1.5 σ) of Asn507 of Nrf2 and nearby atoms.

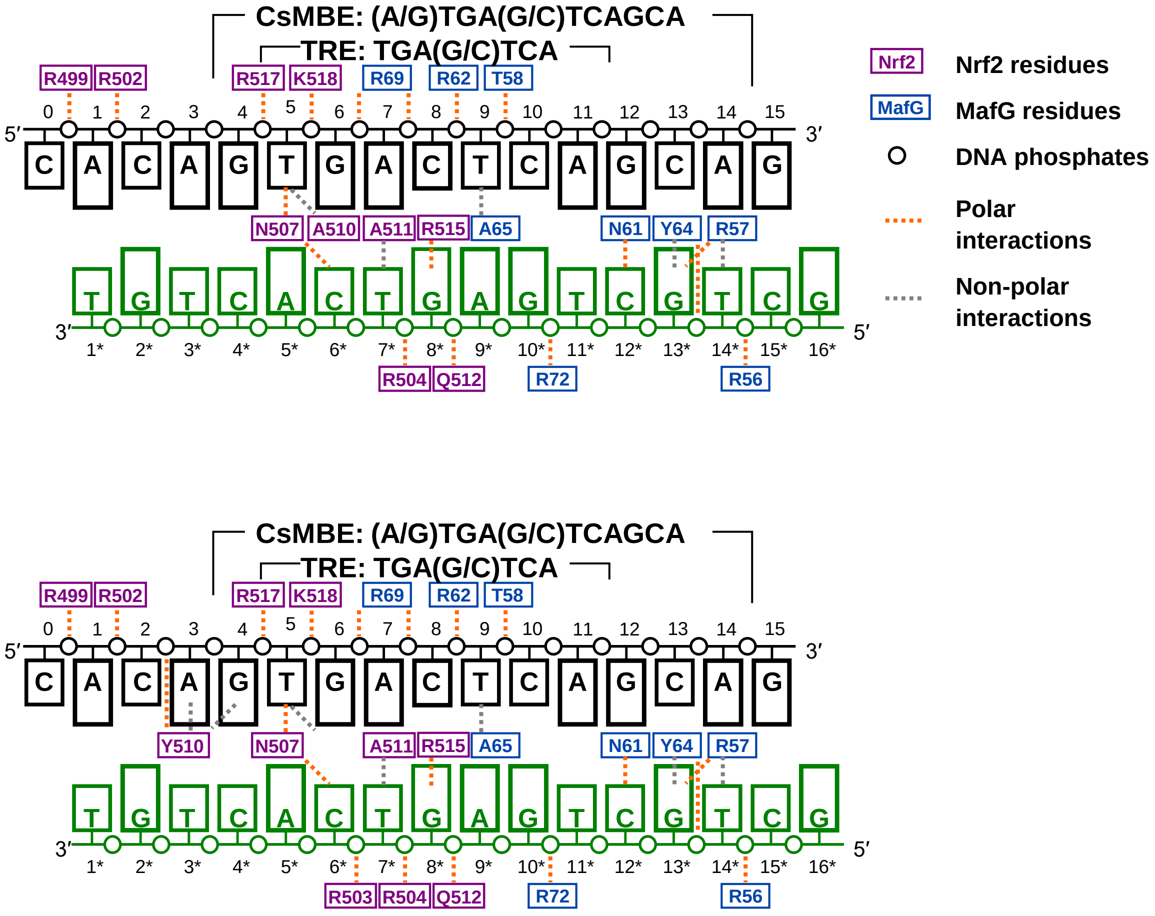

Supplementary Figure S4. Schematic diagram of protein-DNA interactions in the Nrf2-MafG-CsMBE2 and Nrf2 (A510Y)-MafG-CsMBE2 complexes.

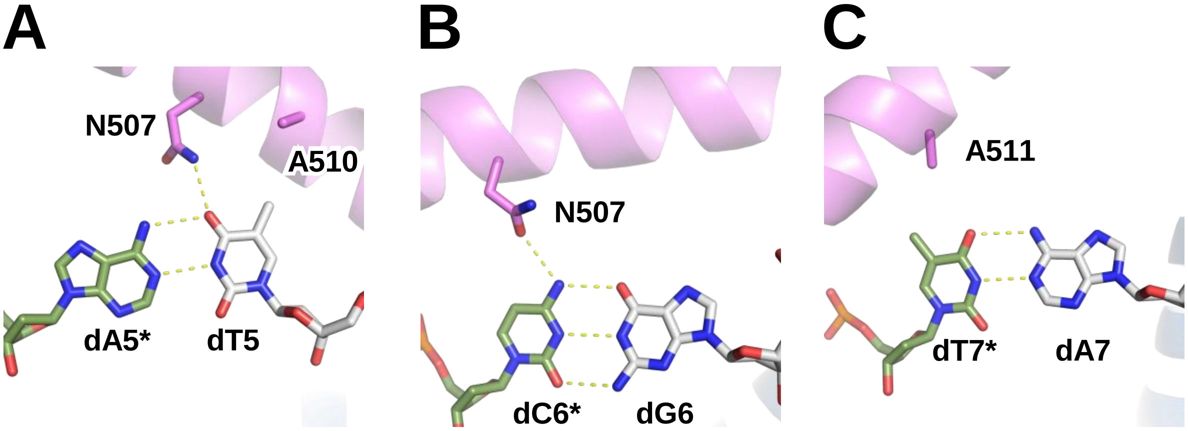

Supplementary Figure S5. Recognition of DNA bases by Nrf2. (**A**) Recognition of dT5. (**B**) Recognition of dC6*. (**C**) Recognition of dT7*.

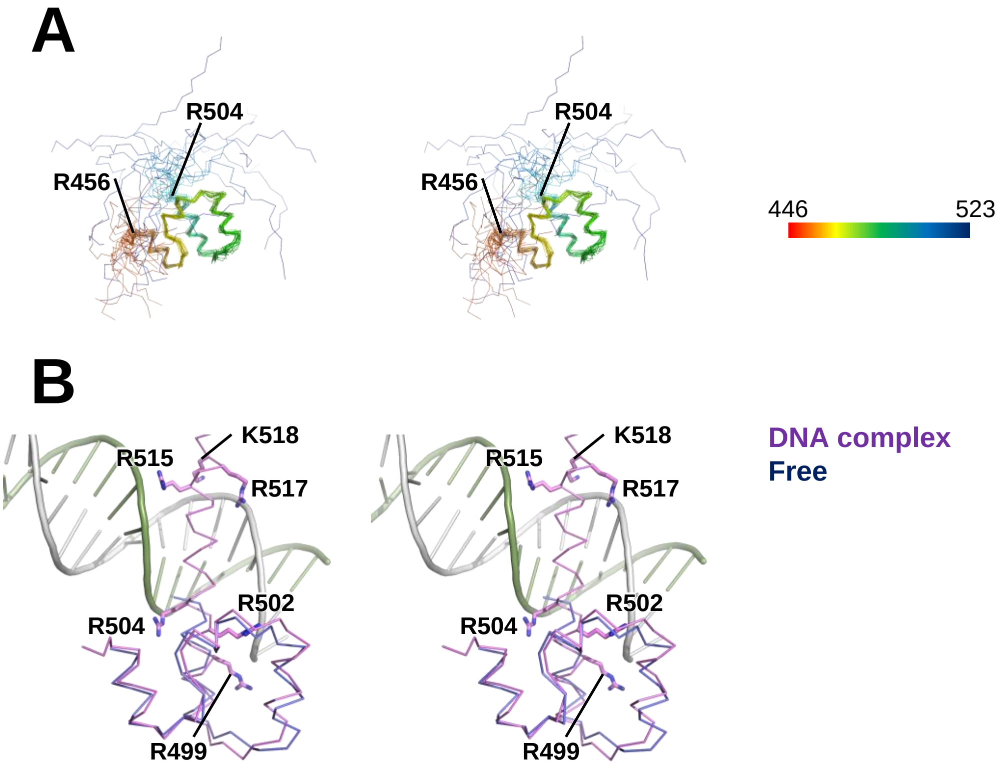

Supplementary Figure S6. Structural comparison between free and complex forms of Nrf2 (stereo view). (**A**) Solution structure of residues 445–523 of free Nrf2 (PDB 2LZ1), showing 20 conformers. Cα trace model are colored according to their residue numbers. (**B**) Superposition of the CNC motifs of the solution structure of free Nrf2 (only residues 456–504 are shown) and the crystal structure of Nrf2 complexed with MafG and CsMBE1. The six arginine residues that contacts the DNA phosphates are also shown.

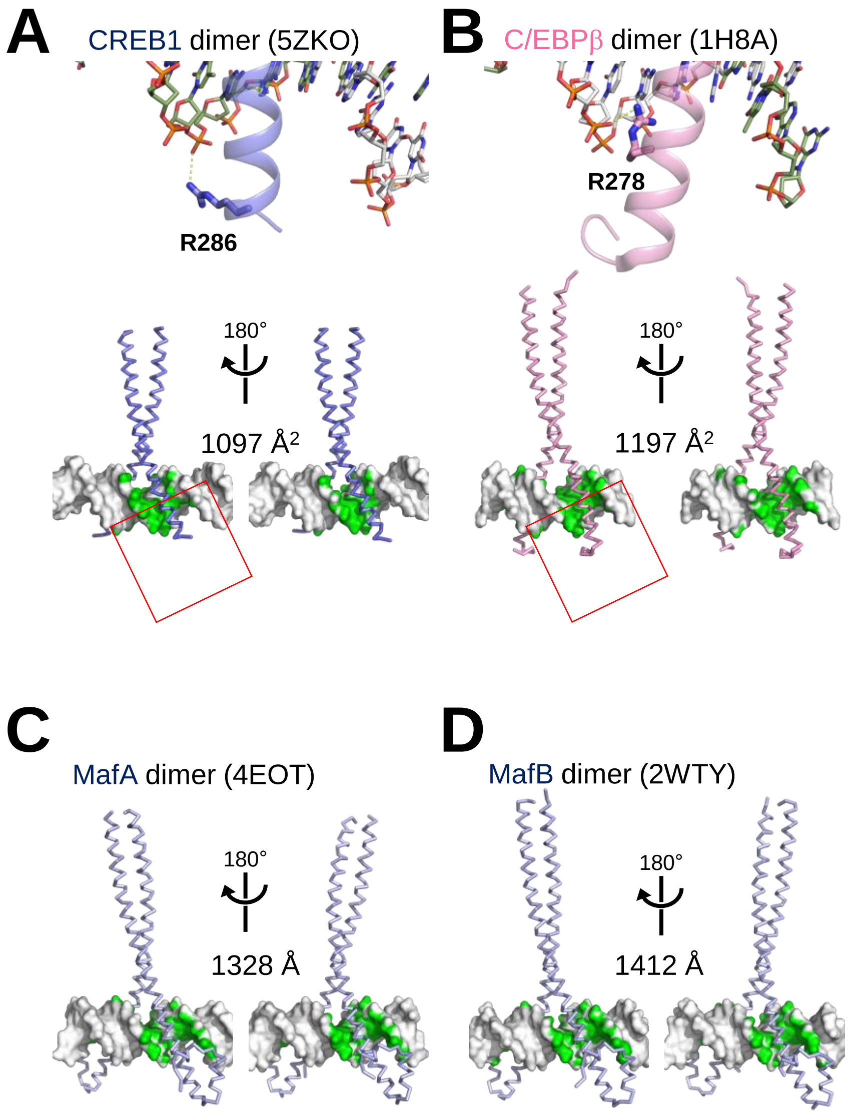

Supplementary Figure S7. Structures of related bZIP proteins bound with DNA. (**A-B**) The interactions between the N-terminal part of the basic regions (top) and DNA contact surfaces colored in green (bottom) by (**A**) the CREB1 homodimer (PDB ID 5ZKO) and (**B**) the C/EBPb homodimer (PDB ID 1H8A). (**C-D**) DNA contact surfaces colored in green (bottom) by (**C**) the MafA homodimer (PDB ID 4EOT) and (**D**) the MafG homodimer.

```
                        10        20        30        40        50        60
                ....|....|....|....|....|....|....|....|....|....|....|....|
                        *    * *                 *  **
Nrf2       492  EAQLALIRDIRRRGKNKVAAQNCRKRKLENIVELEQDLDHLKDEKEKLLKEKGENDKSLH
MafG       46   KEEIIQLKQRRRTLKNRGYAASCRVKRVTQKEELEKQKAELQQEVEKLASENASMKLELD
cJun       247  MESQERIKAERKRMRNRIAASKCRKRKLERIARLEEKVKTLKAQNSELASTANMLREQVA
JunD       263  MDTQERIKAERKRLRNRIAASKCRKRKLERISRLEEKVKTLKSQNTELASTASLLREQVA
cFos       132  LSPEEEEKRRIRRERNKMAAAKCRNRRRELTDTLQAETDQLEDEKSALQTEIANLLKEKE
FosB       150  LTPEEEEKRRVRRERNKLAAAKCRNRRRELTDRLQAETDQLEEEKAELESEIAELQKEKE
C/EBPbeta  266  TVDKHSDEYKIRRERNNIAVRKSRDKAKMRNLETQHKVLELTAENERLQKKVEQLSRELS
CREB1      347  PAEEAARKREVRLMKNREAARECRRKKKEYVKCLENRVAVLENQNKTLIEELKALKDLYC

                ....|..

Nrf2       552  LLKKQLS
MafG       106  ALRSKY
cJun       307  QLKQKV
JunD       323  QLKQK
cFos       192  KLEFIL
FosB       210  RLEFVL
C/EBPbeta  326  TLRNLF
CREB1      407  HKSD
```

Supplementary Figure S8. A sequence alignment of human bZIP proteins. Asterisks indicate Nrf2 residues that bind DNA phosphates.

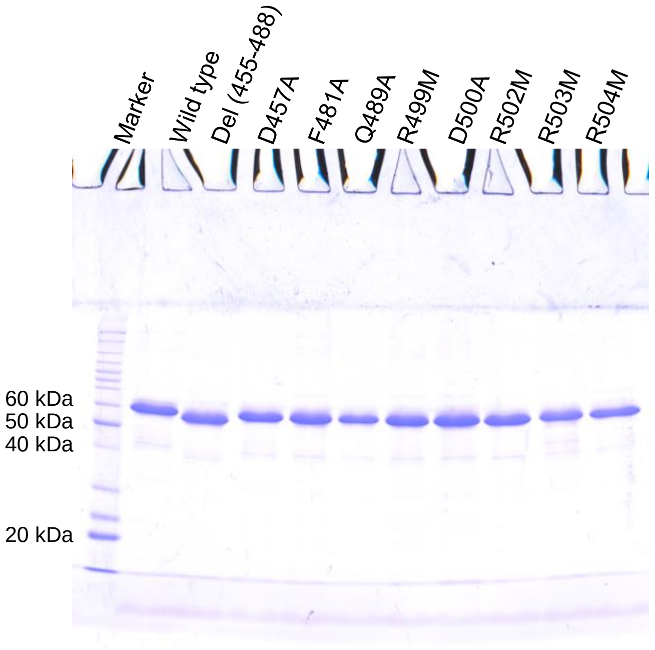

Supplementary Figure S9. Purity of Nrf2 proteins used for EMSA. Three micrograms of purified proteins were analyzed by SDS-PAGE with Coomassie Brilliant Blue staining.

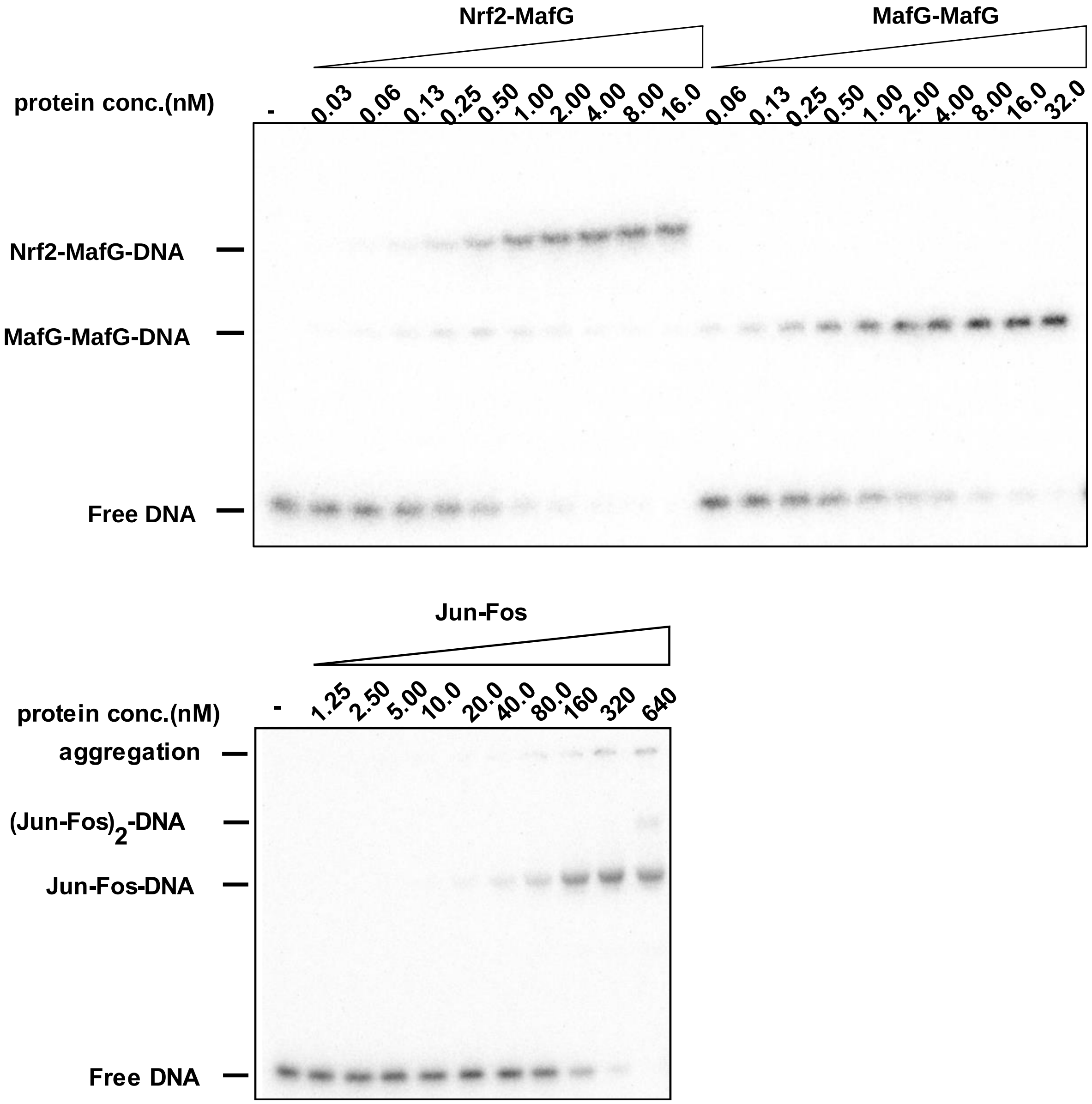

Supplementary Figure S10. Representative gels showing the results of EMSA experiments to measure the affinities of Nrf2-MafG heterodimer, MafG homodimer, and Jun-Fos heterodimer for a DNA fragment containing CsMBE.

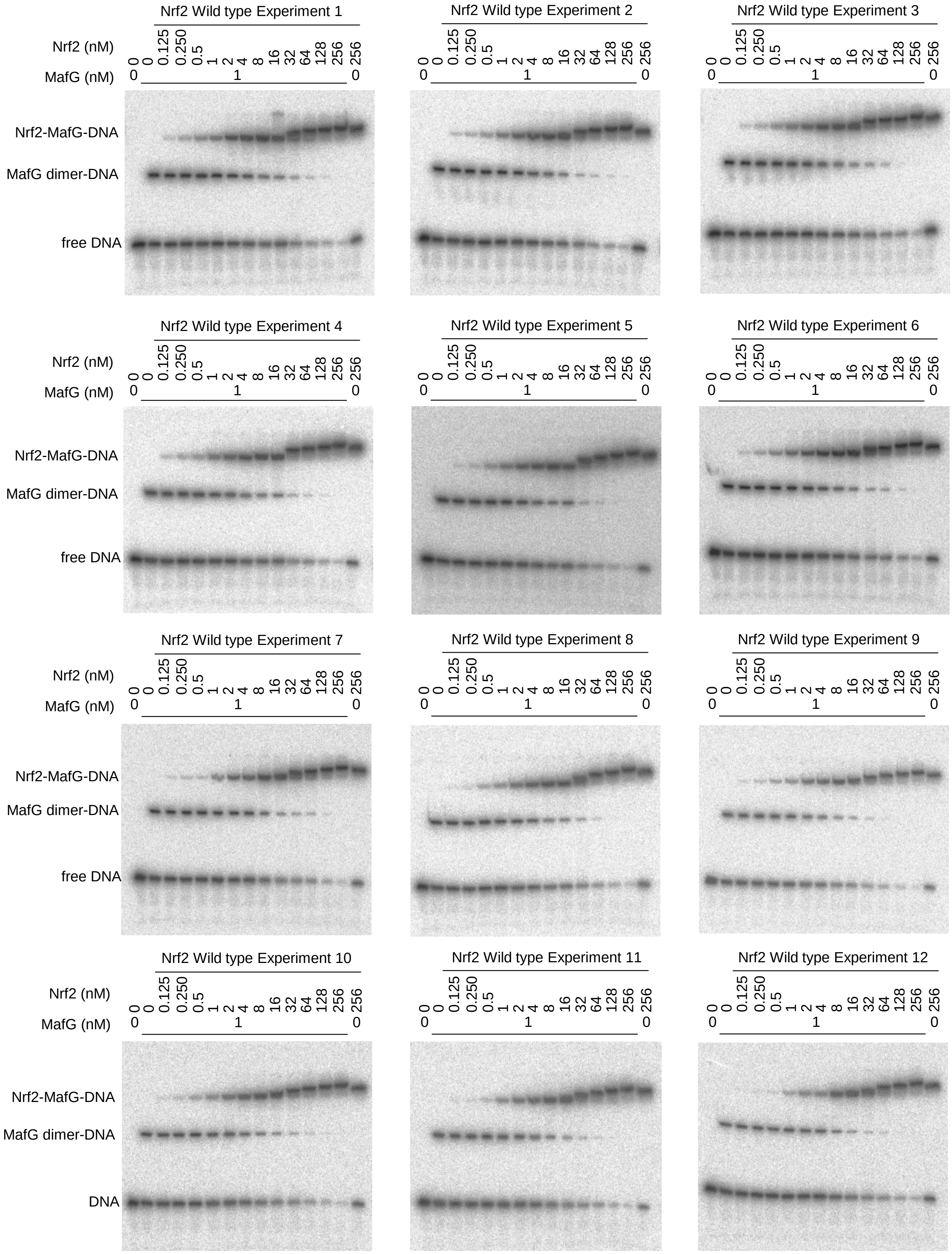

Supplementary Figure S11. Full gel images of competitive EMSA experiments.

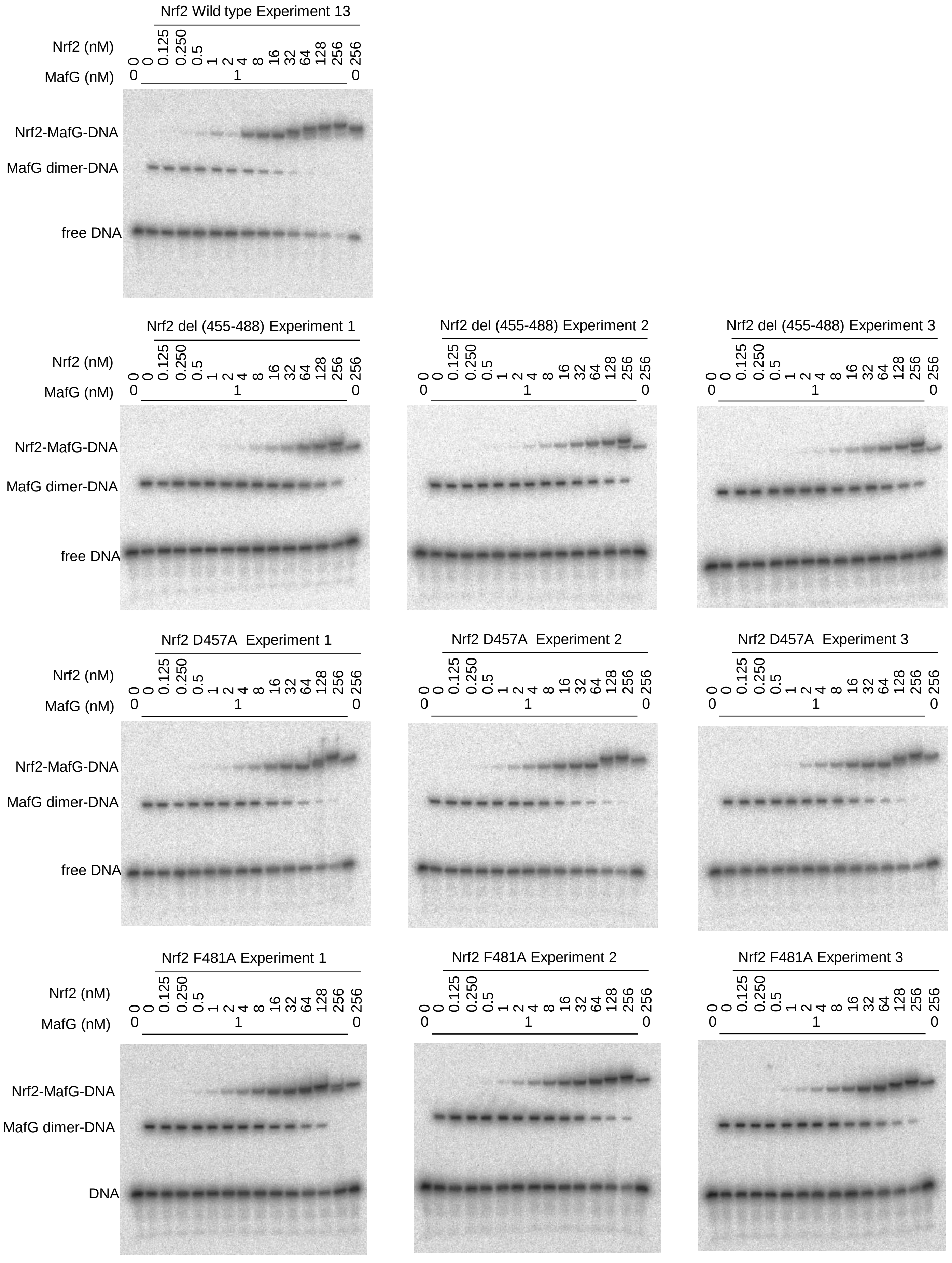

Supplementary Figure S11 (continued).

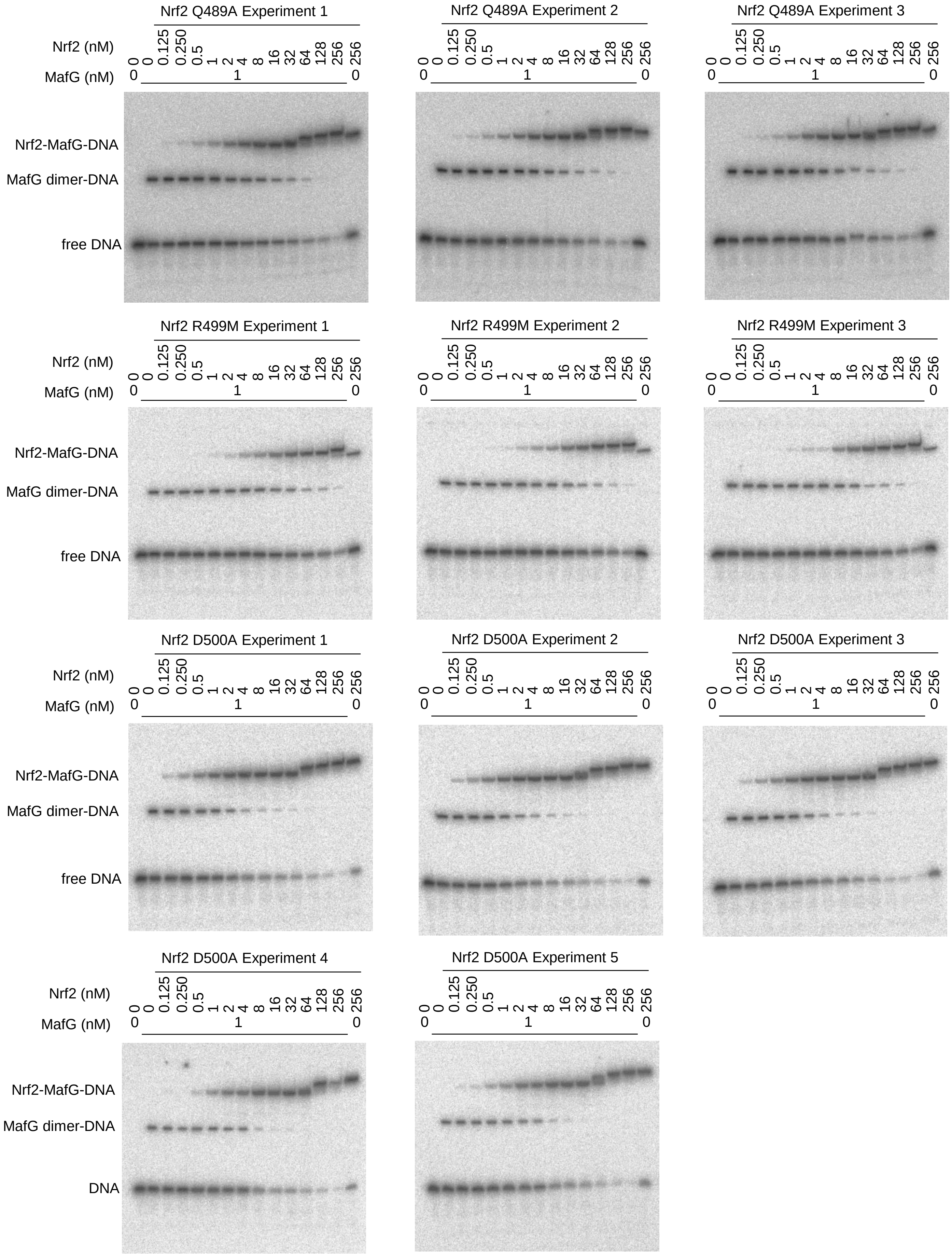

Supplementary Figure S11 (continued).

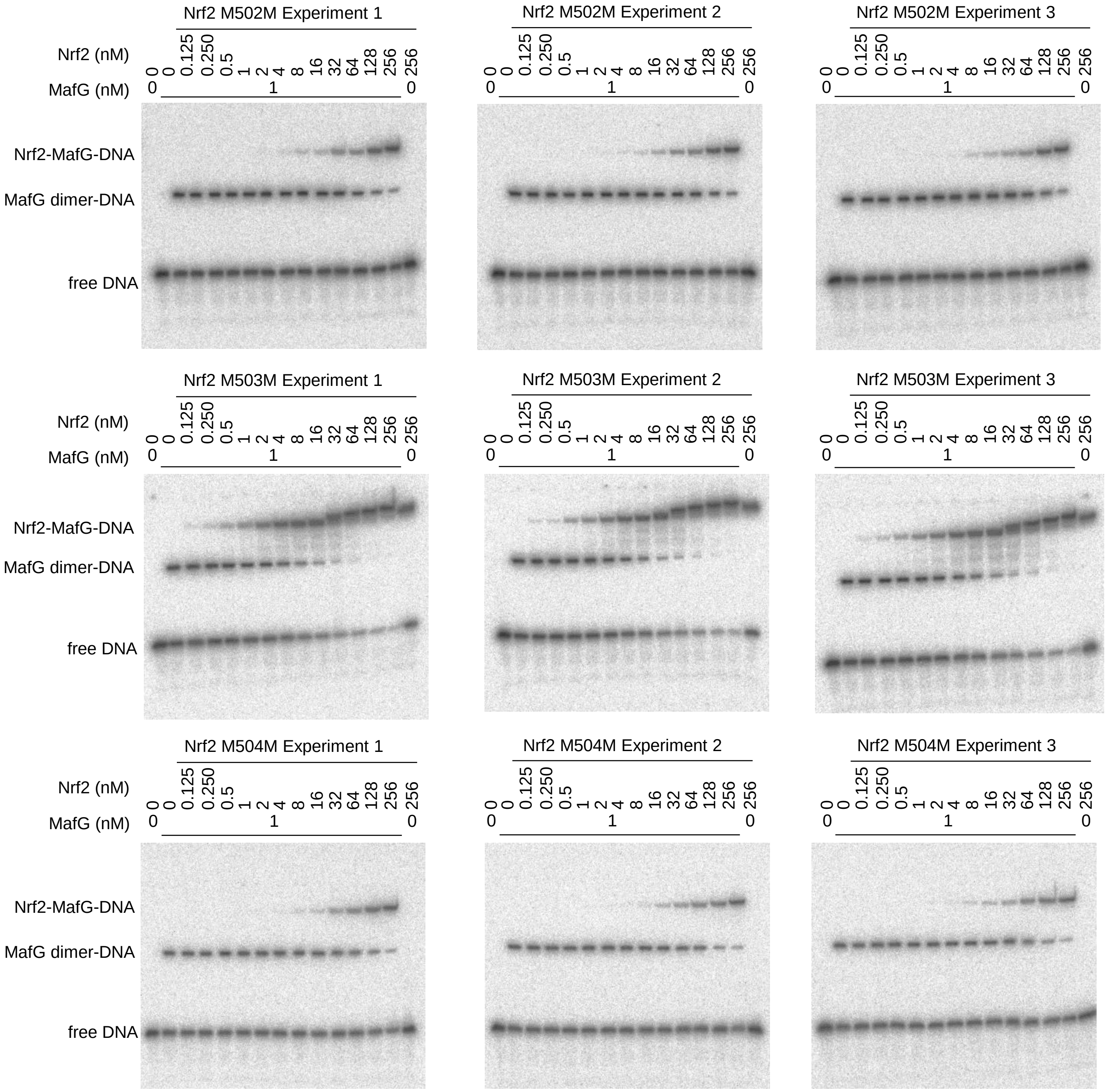

Supplementary Figure S11 (continued).

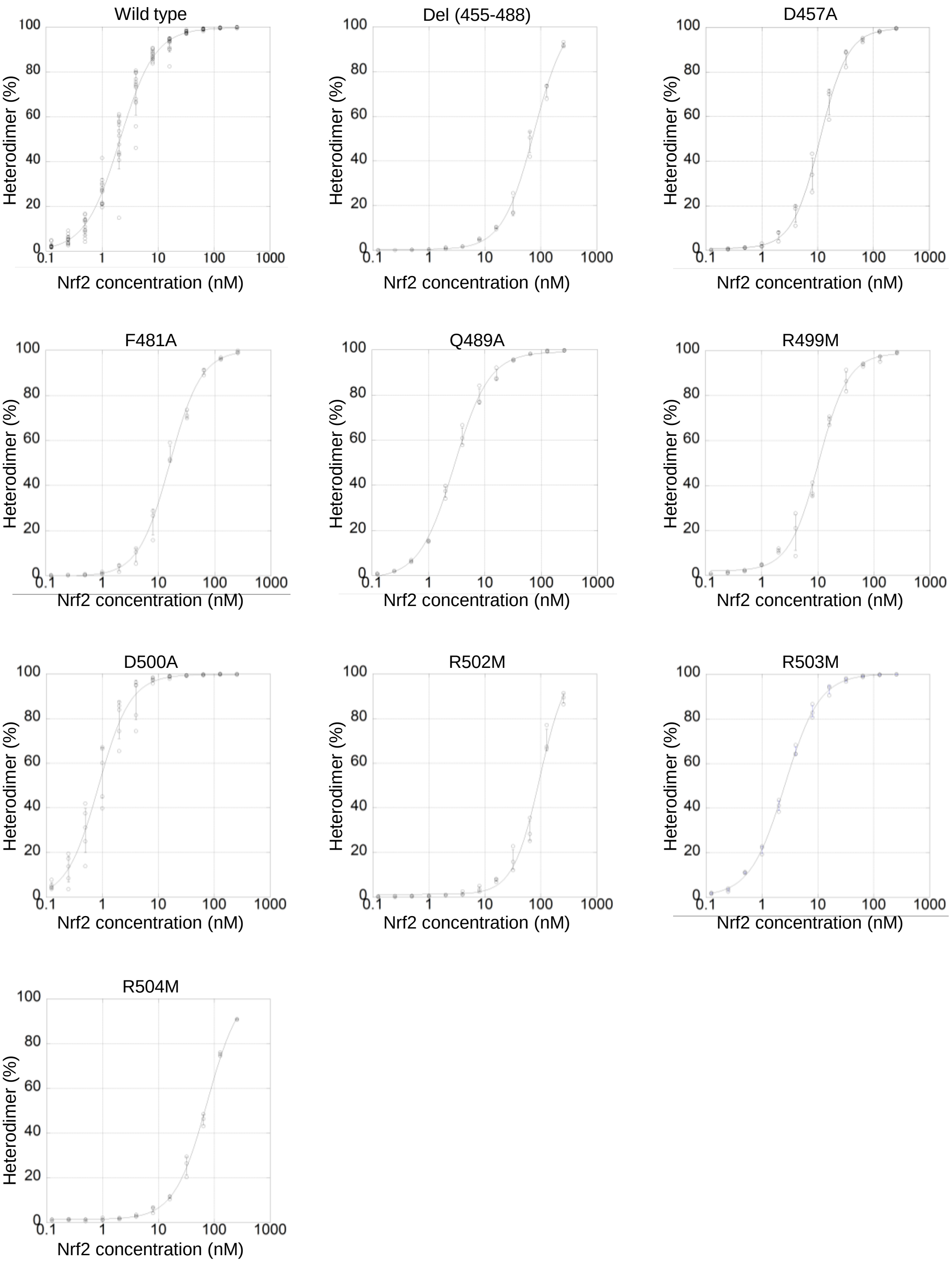

Supplementary Figure S12. Quantification of competitive EMSA. Results of 13 (wild type), 5 (D500A), or 3 (the other mutants) experiments are shown with their standard deviations.

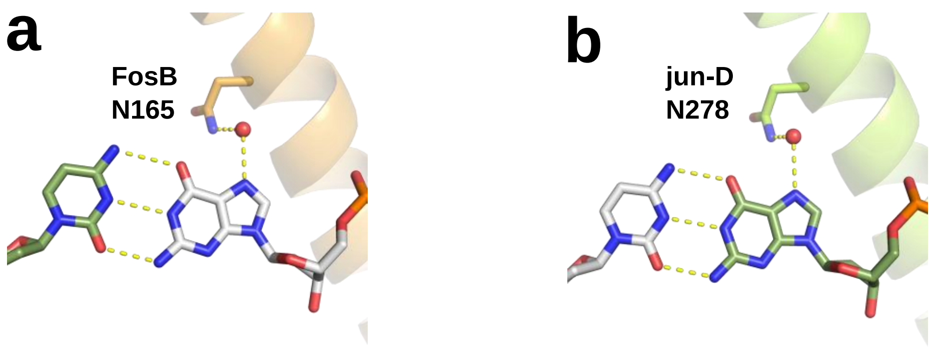

Supplementary Figure S13. Water-mediated recognition of purine at the flanking position by AP-1 proteins (PDB ID 5VPE). (**A**) Guanine recognition by FosB via N165. (**B**) Guanine recognition by Jun-D via N278.

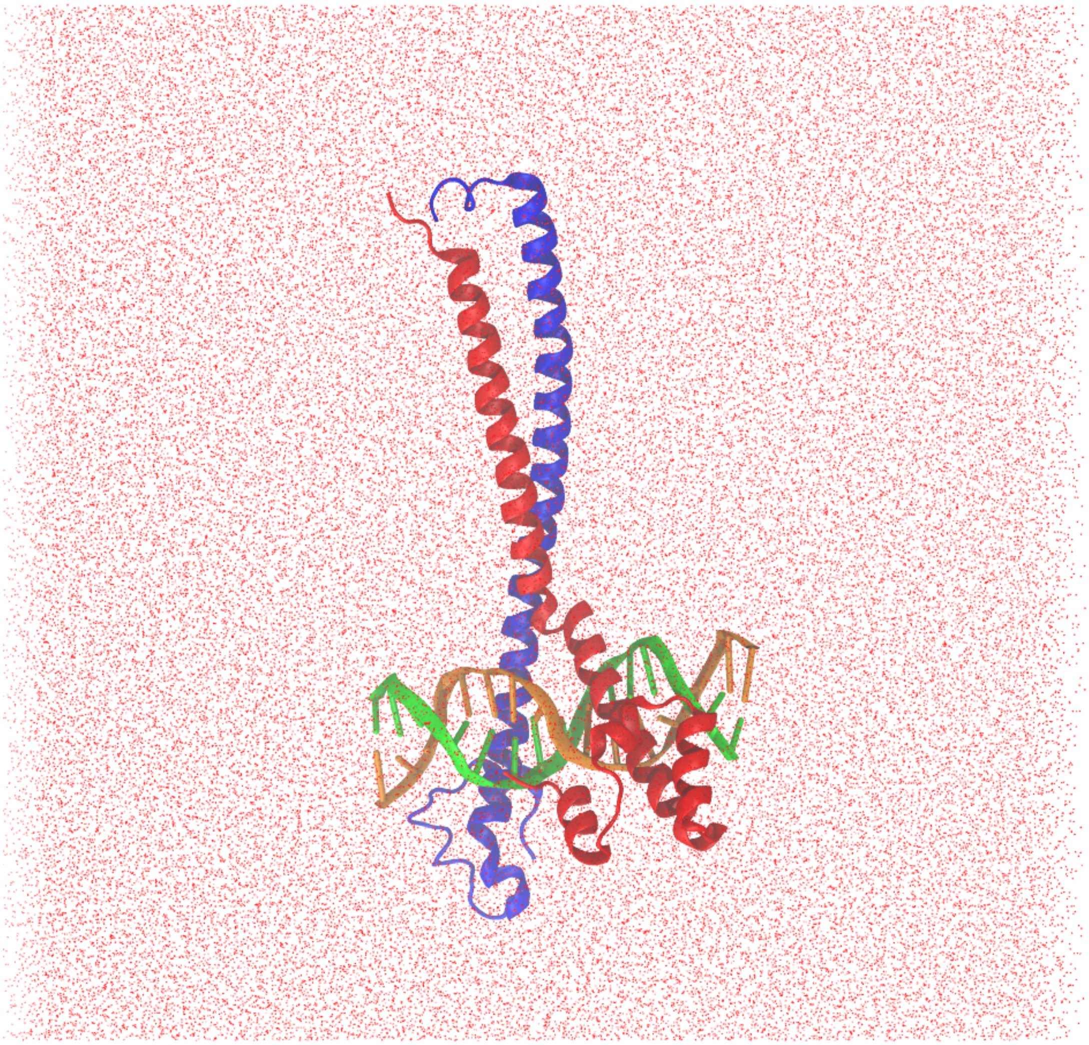

Supplementary Figure S14. A snapshot of an MD simulation for the dA4 system at 30.0 ns (just after the equilibration process).

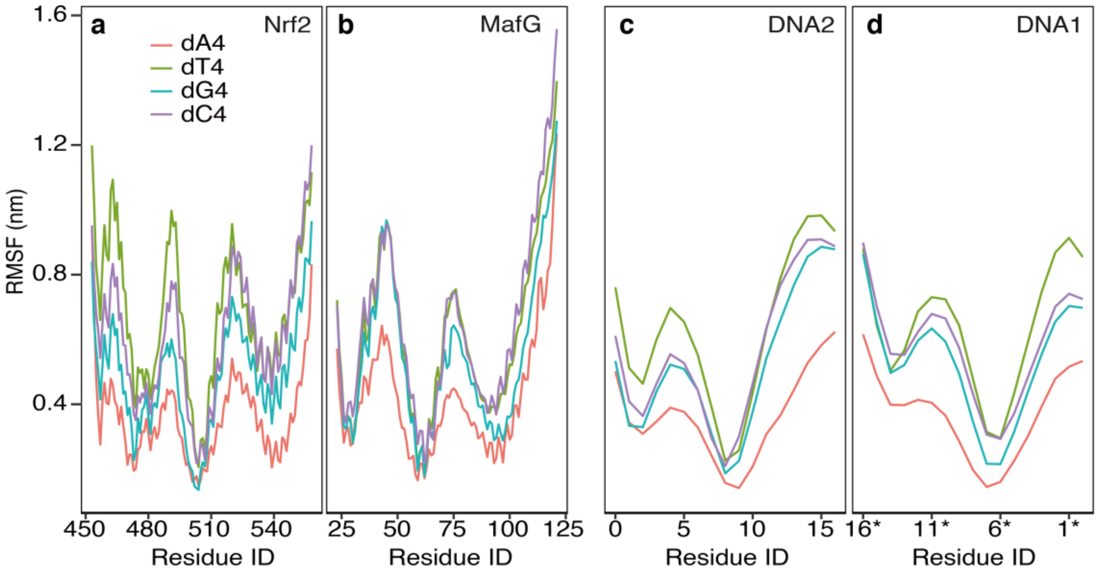

Supplementary Figure S15. MSF values of each residue in the MD simulations. Red, green, cyan, and purple lines indicate the results of the dA4, dT4, dG4, and dC4 systems, respectively.

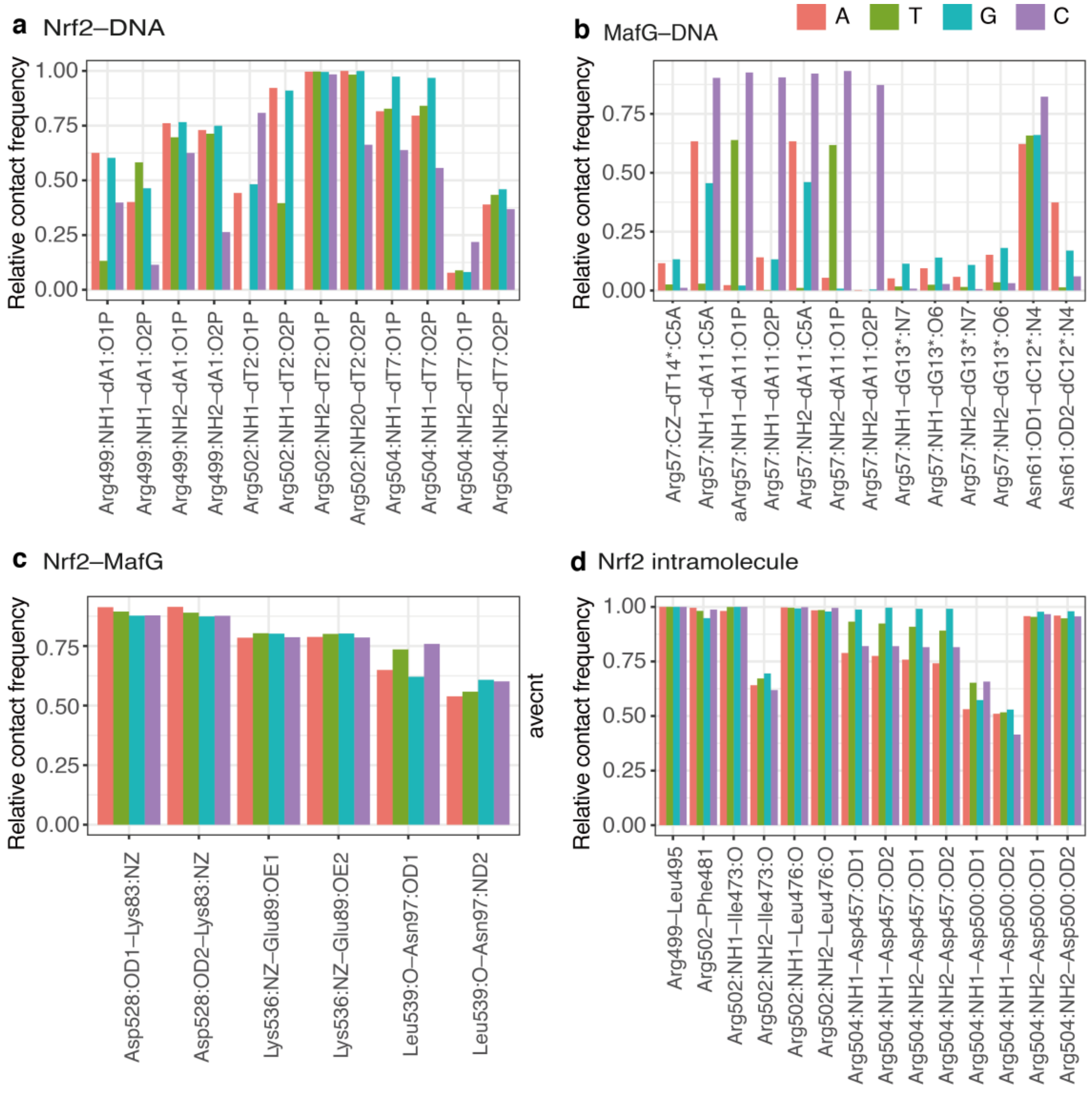

Supplementary Figure S16. Relative contact frequency in the MD simulations. The vertical axis indicates the ratio of simulation times with the contacts, which was defined as the interatomic distance ≤0.5 nm, over four 270-ns production runs for each system. The horizontal axis shows the interacting pairs. The interactions stated in the main text were analyzed. The pair without an atom name analyzed the minimum interatomic distance between two residues. Red, green, cyan, and purple present the dA4, dT4, dG4, and dC4 systems, respectively.

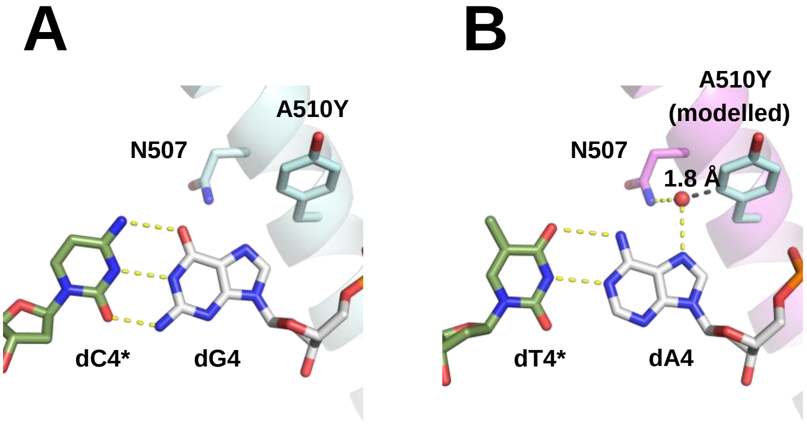

Supplementary Figure S17. Recognition of dG4 by Nrf2(A510Y). (**A**) Recognition of dG4 by A510Y in the crystal structure of the Nrf2(A510Y)-MafG-CsMBE2 complex. (**B**) The modeled A510Y residue based on the crystal structure of Nrf2-MafG-CsMBE1 would clash with the water molecule bridging the N7 atom of dA4 and the N507 sidechain.

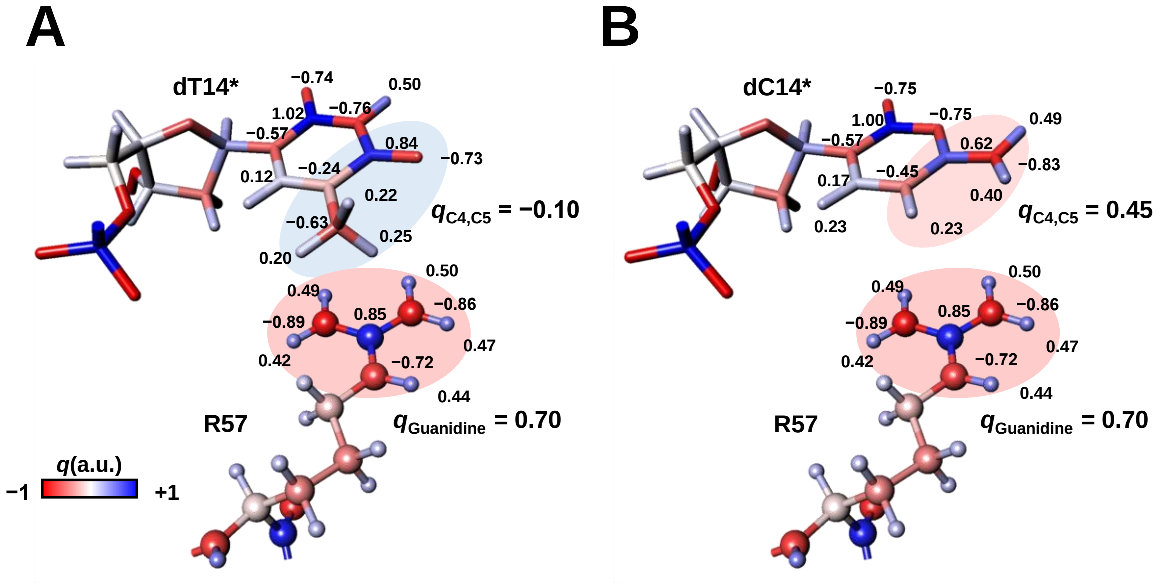

Supplementary Figure S18 . Atomic charges of MafG Arg57 and dT14*/dC14* in the structures of the CsMBE1 and dC14* complexes, as calculated by FMO. (**A**) The CsMBE1 complex. (**B**) The dC14* complex.

Supplementary Table S1. Sequences of recombinant proteins and DNA

Protein	Sequence
Nrf2 (452–560)	*MGSSHHHHHHSSGLEVLFQ*GPHMAHLTRDELRAKALHIPFPVEKIIN<br>LPVVDFNEMMSKEQFNEAQLALIRDIRRRGKNKVAAQNCRKRKLE<br>NIVELEQDLDHLKDEKEKLLKEKGENDKSLHLLKKQLSTL
MafG (21–123)	MGTSLTDEELVTMSVRELNQHLRGLSKEEIVQLKQRRRTLKNRGYA<br>ASCRVKRVTQKEELEKQKAELQQEVEKLASENASMKLELDALRSK<br>YEALQTFARTVAR
His6-Nrf2 (182–560)	MGSSHHHHHHSSGLEVLFQGPHMQDIEQVWEELLSIPELQCLNIEN<br>DKLVETTMVPSPEAKLTEVDNYHFYSSIPSMEKEVGNCSPHFLNAFE<br>DSFSSILSTEDPNQLTVNSLNSDATVNTDFGDEFYSAFIAEPSISNSMP<br>SPATLSHSLSELLNGPIDVSDLSLCKAFNQNHPESTAEFNDSDSGISLN<br>TSPSVASPEHSVESSSYGDTLLGLSDSEVEELDSAPGSVKQNGPKTPV<br>HSSGDMVQPLSPSQGQSTHVHDAQCENTPEKELPVSPGHRKTPFTK<br>DKHSSRLEAHLTRDELRAKALHIPFPVEKIINLPVVDFNEMMSKEQF<br>NEAQLALIRDIRRRGKNKVAAQNCRKRKLENIVELEQDLDHLKDEK<br>EKLLKEKGENDKSLHLLKKQLSTL
His6-MafG (21–123)	*MGSSHHHHHHSSGLEVLFQ*GPHMGTSLTDEELVTMSVRELNQHLRG<br>LSKEEIVQLKQRRRTLKNRGYAASCRVKRVTQKEELEKQKAELQQE<br>VEKLASENASMKLELDALRSKYEALQTFARTVAR
His6-Jun (237–331)	MGSSHHHHHHSSGLEVLFQGPHMGETPPLSPIDMESQERIKAERKR<br>MRNRIAASKCRKRKLERIARLEEKVKTLKAQNSELASTANMLREQV<br>AQLKQKVMNHVNSGCQLMLTQQLQTF
His6-Fos (111–207)	MGSSHHHHHHSSGLEVLFQGPHMVVKTMTGGRAQSIGRRGKVEQL<br>SPEEEEKRRIRRERNKMAAAKCRNRRRELTDTLQAETDQLEDEKSA<br>LQTEIANLLKEKEKLEFILAAHRPACKIP
DNA	Sequence
CsMBE1	Forward: CATGATGAGTCAGCAA<br>Reverse: GTTGCTGACTCATCAT
CsMBE2	Forward: CACAGTGACTCAGCAG<br>Reverse: GCTGCTGAGTCACTGT
EMSA	Forward: CCGCAGTCACAGTGACTCAGCAGAATCTGA<br>Reverse: CTCAGATTCTGCTGAGTCACTGTGACTGCG

Italic residues were removed with HRV3C protease digestion.

Underlines indicate CsMBE motifs.

Supplementary Table S2. Data collection and refinement statistics

Structure	CsMBE1	CsMBE2	A510Y-CsMBE2
PDB ID	7X5E	7X5F	7X5G
**Data collection**			
Space group	*C*2	*C*2	*C*2
Cell dimensions			
a, *b*, *c* (Å)	257.6 54.8 82.3	245.2 54.7 86.0	246.0 55.0 86.2
α, β, γ (°)	90, 96.8, 90	90, 99.2, 90	90, 97.3, 90
Resolution (Å)	50–2.3 (2.38–2.3)	45.92–2.6 (2.76–2.6)	50–2.3 (2.34–2.3)
R_{sym} (%)	7.5 (43.0)	4.1 (64.0)	3.7 (52.1)
$I / \sigma I$	12.2 (2.5)	28.9 (2.9)	34.2 (2.7)
Completeness (%)	99.2 (99.8)	98.8 (94.8)	98.3 (96.5)
CC1/2	99.7 (84.8)	100 (89.2)	100 (89.6)
Redundancy	3.1 (3.1)	7.3 (6.4)	4.6 (4.4)
**Refinement**			
Resolution (Å)	39.76–2.3 (2.38–2.3)	45.27–2.6 (2.69–2.6)	33.08–2.3
No. reflections			
Total	50,646 (4956)	34,187 (3,281)	50,310 (4913)
For R_{free}	5,109 (535)	1,747 (136)	5,070 (498)
R_{work} (%)	21.18 (28.49)	22.60 (33.16)	21.93 (31.83)
R_{free} (%)	24.20 (36.57)	25.87 (39.19)	24.99 (34.88)
No. atoms	4,782	4,557	4,637
Protein	3,295	3,250	3,216
DNA	1,300	1,300	1,300
Ligand/ion	19	0	13
Water	168	7	108
B-factors			
Protein	59.01	91.60	82.84
DNA	58.34	83.50	75.66
Ligand/ion	51.16	-	85.21
Water	46.34	59.51	60.94
RMS deviations			
Bonds (Å)	0.003	0.006	0.003
Angles (°)	0.53	0.76	0.46
Ramachandran			
Favored (%)	99.75	96.98	97.22
Outliers (%)	0	0	0.25

Values in parentheses are for the highest-resolution shells.

Each dataset was collected from a single crystal.

Supplementary Table S3. MD simulation systems

System	# atoms	# water mol.	cell dim (Å)	Sim. Length (ns) [1]	# runs
dA4	274,822	89,927	140.5	300	4
dT4	274,726	89,895	140.5	300	4
dG4	274,728	89,896	140.5	300	4
dC4	274,731	89,897	140.5	300	4

[1] The first 30 ns were omitted for the analyses as equilibration processes.

Supplementary Table S4. Water-mediated hydrogen bonds involving the fourth position of the DNA chain in the MD simulations[1]

DNA		Nrf2		Ratio to keep the hydrogen bonding[2]		
Residue	Atom	Residue	Atom	Average	Max	Min
dA4	H61[3]	Asn507	HD21[4]	0.494	0.609	0.361
dA4	N7	Asn507	HD21	0.520	0.692	0.435
dA4	N7	Asn507	OD1	2.15×10^{-3}	4.22×10^{-3}	7.41×10^{-5}
dA4	H61	Arg503	HH12[5]	5.31×10^{-3}	8.37×10^{-3}	3.63×10^{-3}
dA4	N7	Arg503	HH12	6.11×10^{-3}	1.12×10^{-2}	3.33×10^{-3}
dG4	N7	Asn507	HD21	0.453	0.563	0.327
dG4	N7	Arg503	HH12	2.21×10^{-2}	3.12×10^{-2}	1.16×10^{-2}
dG4	N7	Arg503	HH22	9.70×10^{-3}	1.37×10^{-2}	3.48×10^{-3}

[1] Only hydrogen bonds that are observed to be longer than 1 ns in at least one simulation run are shown. [2] The ratio is calculated as a time length, keeping the hydrogen bonds divided by the simulation length of a production run (270 ns). The average, maximum, and minimum values over the four runs with different initial atomic velocities are shown. [3] Hydrogen of the N6-amino group.

Supplementary Table S5. Values of inter-fragment interaction energy (IFIE) and pair interaction energy decomposition analysis (PIEDA) between MafG Arg57 and nearby residues

The CsMBE1 complex (with dT14*)

Residue	IFIE (kcal/mol)	ES (kcal/mol)	EX (kcal/mol)	CT+mix (kcal/mol)	DI (kcal/mol)	Main component
Asn61	−10.17	−7.05	0.34	−1.23	−2.23	ES
Tyr64	−1.30	−0.55	0.94	−0.42	−1.27	DI
dT14*	−3.98	−2.37	2.13	−0.94	−2.80	DI
dG13*	−62.16	−58.28	9.62	−6.67	−6.83	ES
dC12*	1.89	2.99	0.50	−0.53	−1.07	ES

The dC14* complex

Residue	IFIE (kcal/mol)	ES (kcal/mol)	EX (kcal/mol)	CT+mix (kcal/mol)	DI (kcal/mol)	Main component
Asn61	−10.21	−7.09	0.34	−1.23	−2.23	ES
Tyr64	−1.08	−0.33	0.94	−0.42	−1.27	DI
dC14*	−0.17	1.27	0.40	−0.57	−1.28	DI
dG13*	−62.83	−58.73	9.61	−6.80	−6.91	ES
dC12*	1.88	2.98	0.50	−0.53	−1.07	ES
