# Supplementary material for: Structural basis of transcription regulation by CNC family transcription factor, Nrf2
Source: Nucleic Acids Res. 2022 Dec 1;50(21):12543–57. doi: 10.1093/nar/gkac1102 (PMC9756947; doi:10.1093/nar/gkac1102)
Supplement: gkac1102_Supplemental_Files [file gkac1102_supplemental_files.zip › SupplementaryMovieText.docx]

**Supplementary Movie S1** Motion of the entire structure of the dA4 system for 200–300 ns of the first simulation. The red and blue ribbons are Nrf2 and MafG, respectively, and the green and orange ribbons are DNA chains.

**Supplementary Movie S2** Water-mediated hydrogen bond in the dA4 system. The movie represents 172–190 ns of the third simulation. Asn507 and dA4 residues are shown in the stick model. The water molecules mediating the interactions are shown in sphere models.

**Supplementary Movie S3** Water-mediated hydrogen bond in the dG4 system. The movie represents 162–180 ns of the fourth simulation. Asn507 and dG4 residues are shown in the stick model. The water molecules mediating the interactions are shown in sphere models.

**Supplementary Text S1** The full alignment of 34 CNC family proteins used to create Figure 1C.
